# Two Worlds Colliding: The Interplay Between Natural Compounds and Non-Coding Transcripts in Cancer Therapy

**DOI:** 10.3389/fphar.2021.652074

**Published:** 2021-07-06

**Authors:** Alexandru A. Sabo, Maria Dudau, George L. Constantin, Tudor C. Pop, Christoph-M. Geilfus, Alessio Naccarati, Mihnea P. Dragomir

**Affiliations:** ^1^ Pediatrics 2 (General and Special Pediatrics), Klinikum Stuttgart, Olgahospital, Zentrum für Kinder, Jugend- und Frauenmedizin, Stuttgart, Germany; ^2^ Biochemistry-Proteomics Department, Victor Babes National Institute of Pathology, Bucharest, Romania; ^3^ Department of Cellular and Molecular Biology and Histology, Carol Davila University of Medicine and Pharmacy, Bucharest, Romania; ^4^ Division of Soil Science and Site Science, Thaer-Institute of Agricultural and Horticultural Sciences, Humboldt-Universität zu Berlin, Berlin, Germany; ^5^ Department of Pediatrics, Marie Curie Emergency Clinical Hospital for Children, Bucharest, Romania; ^6^ Division of Controlled Environment Horticulture, Thaer-Institute of Agricultural and Horticultural Sciences, Humboldt-Universität zu Berlin, Berlin, Germany; ^7^ IIGM Italian Institute for Genomic Medicine, Turin, Italy; ^8^ Candiolo Cancer Institute, FPO-IRCCS, Turin, Italy; ^9^ Department of Surgery, Fundeni Clinical Hospital, Carol Davila University of Medicine and Pharmacy, Bucharest, Romania

**Keywords:** natural compounds, non-coding RNAs, cancer, chemotherapy, drug resistance

## Abstract

*Cancer* is a devastating disease and has recently become the leading cause of death in western countries, representing an immense public health burden. When it comes to cancer treatment, chemotherapy is one of the main pillars, especially for advanced stage tumors. Over the years, natural compounds have emerged as one of the most valuable resources for new chemotherapies. It is estimated that more than half of the currently used chemotherapeutic agents are derived from natural compounds. Usually, natural compounds are discovered empirically and an important limitation of introducing new anti-cancer natural products is lack of knowledge with regard to their mechanism of action. Recent data has proven that several natural compounds may function via modulating the expression and function of non-coding RNAs (ncRNAs). NcRNAs are a heterogenous class of RNA molecules which are usually not translated into proteins but have an important role in gene expression regulation and are involved in multiple tumorigenic processes, including response/resistance to pharmacotherapy. In this review, we will discuss how natural compounds function via ncRNAs while summarizing the available data regarding their effects on over 15 types of cancer. Moreover, we will critically analyze the current advances and limitations in understanding the way natural compounds exert these health-promoting effects by acting on ncRNAs. Finally, we will propose several hypotheses that may open new avenues and perspectives regarding the interaction between natural compounds and ncRNAs, which could lead to improved natural compound-based therapeutic strategies in cancer.

## Introduction

### MicroRNAs and Long Non-Coding RNAs in Humans

Almost 2 decades have passed since it has been discovered that about 98–99% of the human DNA is not following the central dogma of molecular biology (i.e., only about 1% of the DNA is actively transcribed into proteins) (The ENCODE Project Consortium, 2012). Part of the non-protein-coding genome, which was initially considered “junk DNA”, was later on discovered to be pervasively transcribed (Palazzo and Lee, 2015) into RNA molecules, and the quest of revealing its functions was started.

Nowadays, a plethora of physiological and pathological functions of the non-coding RNA (ncRNA) are known. Thousands of papers describe the new functions of these previously ignored transcripts (Dragomir M. P. et al., 2018). Based on their size, using an arbitrary selected length of 200 nucleotides, ncRNAs have been divided into small non-coding RNAs (sncRNAs) (<200 nt) and long non-coding (>200 nt) (lncRNAs). The most studied class of ncRNAs is a particular subtype of sncRNAs called microRNAs (miRNAs). MiRNAs, approximately 22 nt in length, typically bind the 3’ untranslated region (3’UTR) of mRNAs, further inhibiting their translation into proteins. Worth mentioning is also that most mRNAs are targeted by multiple miRNAs and each miRNA can target several mRNAs, forming complex regulatory networks (Dragomir M. et al., 2018). An additional layer of complexity to these regulatory networks is the involvement of lncRNAs (or other non-coding genes) that can bind and sponge miRNAs, interfering with their canonical function of mRNA suppression. Hence, it seems that there is a pool of miRNAs that can bind both other ncRNAs and mRNAs, and everything depends on the stoichiometry of these molecules. Interestingly, this observation was first made in plants. In *Arabidopsis thaliana*, high levels of the non-coding transcript *IPS1* lead to the sequestration of miR-399, which consequently induces the up-regulation of its target, PHO2 mRNA (Franco-Zorrilla et al., 2007). This interplay is perceived as a unifying theory, connecting the non-coding world and the coding world, being plausible especially in plants (Paschoal et al., 2018).

In the past decade, we have also witnessed some atypical miRNA mechanisms of action. For example, some miRNAs bind to their target mRNAs, inducing their translation rather than inhibiting it, while other miRNAs activate Toll-like receptors (TLRs) (Dragomir M. P. et al., 2018). Therefore, an altered miRNA transcriptome will generate an altered proteome. Moreover, miRNAs, in a similar way as small hormones, can function in intercellular signaling pathways in a paracrine or endocrine fashion, being packed into exosomes or bound to proteins/lipids for short and long-distance transport (Pardini and Calin, 2019). These “traveling miRNAs” can be altered in various pathological states and reflect most of the circulating ncRNA transcriptome.

A special subtype of sncRNAs are those arising from introns, which act as non-canonical miRNAs and were therefore termed “mirtrons”. Deregulation of these mirtrons is involved in various human pathologies. Also, in plants, these molecules might have miRNA regulatory roles. MirtronDB is an initiative of collecting available data on these molecules (Da Fonseca et al., 2019).

LncRNAs, as mentioned, are >200 nt in length, but can also be several kilobases long. They have a complex secondary structure including double stem loops and cloverleaf elements, being polymorphic in form, which enables them to carry out a wide range of functions (Novikova et al., 2012). LncRNAs are capable of regulating gene transcription, directly binding proteins and RNA molecules, acting as post-transcriptional regulators and signaling regulators (Statello et al., 2021). LncRNAs also influence chromatin remodeling (Dragomir et al., 2020a). Surprisingly, in recent years, it was observed that numerous lncRNAs, circular RNAs and a few miRNA precursors are translated into functional micropeptides that play a role in immunity, development and cancer (Dragomir et al., 2020b; Othoum et al., 2020). From a phylogenetic standpoint, lncRNAs can be extremely conserved, being termed “transcribed ultraconserved regions” or they can be primate-specific, often harboring short repetitive regions called pyknons (Rigoutsos et al., 2017; Dragomir et al., 2020a). Over the years, other forms of ncRNAs with various functions have been described and new ones are currently under investigation. For more information about other classes and the main functions of ncRNAs, we refer to Cech and Steitz (2014) and Pardini et al. (2019).

The role of ncRNAs in cancer is one of the most extensively studied fields of non-coding transcripts. Currently, we can consider that ncRNAs play functional roles in all 12 canonical hallmarks of cancer, but also in emerging cancer traits like oncogenic neurogenesis (Amit et al., 2020). For example, the super-oncogenic miR-21 is overexpressed in several types of cancer and induces proliferation, migration, invasion, and metastasis (Javanmardi et al., 2017) and the super-tumor suppressor miR-34 family members are dysregulated in a plethora of cancers and are known to inhibit metastasis by blocking epithelial-mesenchymal transition (EMT) (Zhang L. et al., 2019). An important role in deciphering the function of ncRNAs are the multiple databases that provide crucial information regarding their interacting molecules, structure, function etc. (Maracaja-Coutinho et al., 2019). For example, a better understanding of the role of ncRNAs in cancer will stem from the analysis of their secondary structure, which will provide additional mechanistic insights. A tentative to provide a curated resource on this topic has been recently done by the RNAcentral Consortium which provides the largest database regarding ncRNA spatial organization for >13 million molecules (RNAcentral Consortium et al., 2021).

**FIGURE 1:**
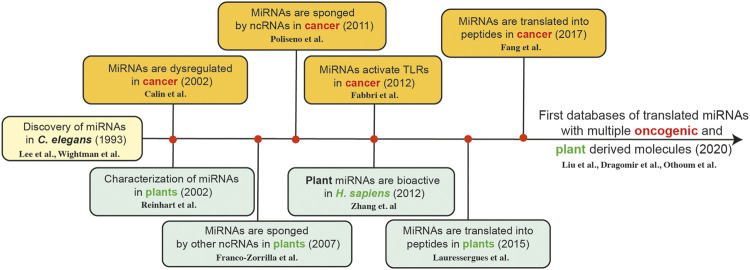
The timeline of miRNA discoveries is related plant sciences and oncology; providing a new understanding about potential new plant based therapeutic tools in cancer therapy [Lee et al. (Lee et al., 1993), Wightman et al. (Wightman et al., 1993), Calin et al. (Calin et al., 2002), Reinhart et al. (Reinhart et al., 2002), Franco-Zorilla et al. (Franco-Zorrilla et al., 2007), Poliseno et al. (Poliseno et al., 2010), Fabbri et al. (Fabbri et al., 2012), Zhang et al. (Zhang et al., 2012), Lauressergues et al. (Lauressergues et al., 2015), Fang et al. (Fang J. et al., 2017), Liu et al. (Liu H. et al., 2020), Dragomir et al. (Dragomir et al., 2020b), and Othoum et al. (Othoum et al., 2020)].

While we continue to understand more and more about the role of specific ncRNAs in different types of cancer, from a translational perspective, these molecules have a promising future as diagnostic biomarkers and therapeutic tools in cancer (Petrescu et al., 2019). Additionally, we observe that the timeline of miRNA discoveries is related to the plant world and oncology (Dragomir M. P. et al., 2018; Li J. et al., 2019), providing a new understanding about potential new plant based therapeutic tools in cancer therapy (Figure 1).

### From Plants to Chemotherapy

Serendipity, which represents the “finding of interesting or valuable things by chance”, together with recurrent efforts, are the best words to historically describe the discovery of most chemotherapeutic compounds (Mann, 2002). In the first half of the 20th century, the main cancer treatment dogma was based on radiation and surgery. Research into usage of chemicals as anti-cancer agents emerged based on empirical observations. For example, spilled mustard gas from a bombed ship in Bari in World War II induced extreme leukopenia, as observed in depleted bone marrow and lymph nodes of exposed individuals. At the same time, the dietary effects of folic acid were discovered, subsequently leading to the development of folate antagonists as therapeutic agents. Such discoveries opened the first avenues to chemotherapy. Among them, the most notable is methotrexate. In addition, the discovery of the effects of thiopurines and 5-fluorouracil (5-FU) in cancer induced a shift of paradigm in cancer treatment, marking the transition from surgical treatment to the modern “chemotherapy”. An important landmark that needs to be mentioned was the foundation of the “*Cancer* Chemotherapy National Service” in 1955 in the United States, which started a new era of substance research, where natural compounds where screened and then tested (DeVita and Chu, 2008).

Plants are the main source of natural compounds discussed in this review. We will use the term “phytochemicals” to refer to plant secondary metabolites that fulfill a broad range of functions e.g., in the defense against herbivores and pathogens, in the interaction with pollinators, in plant-plant chemical communication or in the protection against high light or UV radiation, drought or other abiotic threats. Since plants cannot actively change their location, they have to overcome inconvenient conditions rather than switching to more convenient places. Being embedded in such complex ecological relationships, plants evolved a staggering diversity of chemical compounds enabling them to optimize their sessile lifestyle to withstand specific pressures. This gave rise to a huge source of molecules, including some with potential health benefits to humans. Knowing that compounds with potential uses in cancer therapy are the result of a stress-induced shift in plant metabolism toward a defense reaction, appropriate techniques can be used in controlled horticulture and agriculture to enrich crops with these bioactive compounds. Exposing the plant to a short and controlled stress treatment will stimulate biosynthesis of these compounds without reducing yield (Geilfus, 2019).

The structure and relative quantity of phytochemicals vary not only between species but even among different populations of the same species and tissues therein (Moore et al., 2014). In the context of medical research generally and cancer research specifically, plant genetic diversity and ecological diversity, the main drivers of chemical diversity in secondary metabolites, are thus invaluable resources.

With the development of adequate models for testing these compounds, more and more natural products from a variety of sources, belonging to a wide range of chemical classes have been screened in order to find new agents. This way, the functions of phytochemicals previously used in traditional medicine have been explained. These findings laid the ground for improvement, as starting from the original molecules found in nature, more potent and effective chemical analogs have been synthesized by techniques such as combinatorial chemistry (Cragg and Pezzuto, 2016).

Since the second half of the 20th century, the approach on oncologic treatment has changed, as more and more natural products known from traditional medicine were shown to be effective in treating or at least slowing down cancer. The precursors of the podophyllins which are used today, with etoposide and teniposide being the most representative ones, stem from the mayapple plant (*Podophyllum peltatum* L.), which Native Americans have been using in the treatment of skin warts. Likewise, *Catharanthus roseus* (L.) G. Don (syn. *Vinca rosea* L.), a plant that has been used as a remedy in traditional Asian medicine, yields potent anti-cancer drugs nowadays. The isolation of paclitaxel from the Pacific yew tree (*Taxus brevifolia* Nutt.) and, later on, of the more efficient analog docetaxel from the European yew (*Taxus baccata* L.), revolutionized the standard approaches of oncologic care, as they have been shown to be efficient against breast, pancreatic and lung cancer. Likewise, the topoisomerase inhibitors topotecan and irinotecan are also of plant origin, derived from *Camptotheca acuminata* Decne., a tree species native to China, and are part of standard treatment protocols of different cancers. At the same time, research initiatives also focused on discovering chemotherapeutic agents from other sources besides plants. For example, two of the chemotherapeutical agents frequently used–mitomycin and doxorubicin–are derived from microbial sources. Likewise, cytosine arabinoside (cytarabine), commonly used in pediatric cancers, is derived from a marine sponge (Mann, 2002).

While for many well-stablished natural compounds the main mechanism of action in cancer is already known, for newer compounds the mechanisms still need to be elucidated. Additionally, alternate mechanisms are being investigated. New discoveries over the last 2 decades proved that older and newer natural products convey their functions in cancer treatment via modulating ncRNA expression (Izzotti et al., 2012). In this review, we sum up recent literature focused on investigating the effects of natural compounds/phytochemicals through ncRNAs on different cancers, discussing both cancers of the adult age and pediatric cancers. From this, we have identified both open fields for research and putative phytochemicals whose potential for human health promotion might be worth pursuing in depth. As most studies regarding phytochemicals and cancer have focused on miRNAs and lncRNAs, we will only focus on these two classes of ncRNAs.

### Sources and Classes of Natural Compounds

A natural compound is any organic product that is synthesized by a living organism. As stated above, the main sources of natural compounds are terrestrial plants, marine macroorganisms and microorganisms (both terrestrial and marine). In this section, chemicals derived from each of these sources will be briefly addressed.

In terms of their role in human diets, phytochemicals are defined as bioactive substances found in fruits, leaves, stems, tubers, roots and other parts of the plant, providing beneficial health effects. Unlike other human nutrients such as proteins, fats or minerals, most phytochemicals are not indispensable for human life, but, among other beneficial properties, they have considerable value in cancer prevention, and as adjuvants in cancer therapy (Gullett et al., 2010; Sak, 2012).

Beside the terrestrial plant kingdom, marine life is also a major source for a variety of bioactive agents. These can be extracted from marine macroorganisms like tunicates, sponges, soft corals, and mollusks. Noteworthy examples would be cytarabine and vidarabine, two agents widely used in chemotherapy (Montaser and Luesch, 2011; Victor and Sharma, 2015).

Likewise, microbes produce a wide range of potentially useful compounds. Bacterial proteins and peptides and, more recently, chemicals isolated from marine sediment bacteria include promising natural compounds that show an anti-proliferative activity and could be employed as anti-cancer agents. Antibiotics, bacteriocins, enzymes, non-ribosomal peptides (NRPs) and toxins are the main groups of bacteria-derived chemicals (Karpinski and Adamczak, 2018).

Natural compounds can be classified into different groups based on their chemical structure and biological characteristics including phenolic compounds, terpenes, carotenoids, saponins, alkaloids and organosulfur compounds, among others (Budisan et al., 2017). Table 1 offers an overview of the chemical classes and subclasses encompassing the natural products discussed in this review, which are almost exclusively of plant origin. It should be stressed that this is not an exhaustive table attempting to categorize the entire spectrum of known natural compounds, but only a selection relevant in the context of ncRNA-mediated anti-cancer effects. The reader may also acknowledge that there are also disciplines that favor a different way of classification or grouping. In the next section, we will present recent findings about specific natural compounds in the context of ncRNA modulation and cancer therapy, following the order used in Table 1.

**TABLE 1:** Major classes and subclasses of organic chemicals, and their specific natural compounds, whose potential role in cancer therapy has been summarized in this review. N/A, not available.

Major compound classes	Subclasses
Phenolic compounds	Phenolic acids and other monophenols
	Flavonoids: Flavones, flavanones, isoflavones, flavanols, chalcones
	Curcuminoids
	Stilbenoids
	Tannins: Proanthocyanidins
	Related compounds: Lignans, flavaglines
Terpenoids and meroterpenoids	Monoterpenoids, diterpenoids and triterpenoids
	Carotenoids
	Saponins
	Polyprenylated acylphloroglucinols
	Cannabinoids
Alkaloids	N/A
Polysaccharides	N/A
Organosulfur compounds	N/A

## Phenolic Compounds

Plant phenolic compounds are secondary metabolites bearing structures with one or more hydroxyl groups attached to at least one aromatic ring. They are generally known for their antioxidative properties, with the number and position of the hydroxyl groups leading to a variation in their bioactivity, e.g., in the antioxidative potential. In plants, they represent precursors of cell wall components and fulfill a multitude of other functions including the response to different biotic and abiotic stressors. For instance, phenolic substances are known to have repellent properties against herbivores, diminish herbivore digestive ability, protect against fungal and bacterial infections and are involved in the response to wounding, drought, salinity and modified or stressful light exposure (Bhattacharya et al., 2010; Geilfus, 2019).

Very often, phenolic compounds extracted from plants are referred to as “polyphenols”, both in commercial and in scientific communication. However, this term is sometimes misused to even include compounds featuring a single hydroxylated aromatic ring, which would be correctly referred to as monophenols. In this review, we use definitions and classifications proposed by Quideau et al. (2011) as guiding principles for presenting plant phenolic compounds in a systematic fashion. This can be a challenging task, since common classifications of plant phenolic compounds based on biogenetic aspects yield categories that cannot be globally considered monophenols or polyphenols, despite this being a very common practice. For instance, flavonoids, curcuminoids and stilbenoids are commonly referred to as “polyphenolic compounds”, even though these classes also contain compounds with less than two hydroxylated benzene rings. For this reason, we will discuss these classes of phytochemicals directly under the umbrella term of “phenolic compounds”, while only using the term “polyphenols” when all criteria proposed by Quideau et al. (2011) are met. These authors define polyphenols as “*plant secondary metabolites derived exclusively from the shikimate‐derived phenylpropanoid and/or the polyketide pathway(s), featuring more than one phenolic ring and being devoid of any nitrogen‐based functional group in their most basic structural expression.*”

### Phenolic Acids and Other Monophenols

Phenolic acids are monophenolic compounds and are some of the most abundant dietary antioxidants in everyday human diets, consumed as fresh or processed fruits, e.g., raspberries, blueberries, cranberries, apples, and grapes (including wine) and also present in tea and coffee (Manach et al., 2004). This class of compounds is mainly divided in two subgroups: hydroxybenzoic acids and hydroxycinnamic acids (Velderrain-Rodríguez et al., 2014; Kumar and Goel, 2019). Like other phenolic compounds, phenolic acids do not only display antioxidative properties upon human consumption, but also show antimicrobial, antiviral, anti-mutagenic, or anti-tumorigenic activity (Shahidi and Yeo, 2018; Kaurinovic and Vastag, 2019; Valanciene et al., 2020). As intermediate substrates of the plant-intrinsic phenylpropanoid pathway, some phenolic acids are chemical precursors of many other monophenolic or polyphenolic phytochemicals presented here.

**Gallic acid** is a trihydroxybenzoic acid distributed in a wide range of plants such as sumac species (*Rhus* spp.), oaks (*Quercus* spp.) and others. Moreover, it is present in wine. During vinification, gallic acid travels from the oak barrel into the wine (Giménez Martínez et al., 2001). It is so stable, that it survives the fermentation and ripening process and year-long storage. A study on the effects of gallic acid on chondrosarcoma cells showed that this compound reduces viability, inhibits migration and induces apoptosis. Bcl-2 levels were decreased and Bax levels were increased and caspase-3 and -9 activation was enhanced. Moreover, miR-518b was up-regulated in chondrosarcoma cells treated with gallic acid, suggesting its implication in apoptosis and inhibition of migration, but no molecular pathway was directly described (Liang et al., 2014).

**Salidroside**, a glucoside of the monophenolic compound tyrosol extracted from the golden root plant (*Rhodiola rosea* L.), was used to treat nephroblastoma tumor cell lines. Apoptosis was promoted, cleaved caspase -3 and -9 levels were elevated, p53 and p21 expression was increased and decreased expression of vimentin, matrix metalloproteinase 2 (MMP-2) and cyclin D1 was observed. The phosphorylation levels of p65, Iκ-Bα, PI3K, AKT and mTOR were increased in treated cells, while transfection with miR-891b up-regulated cyclin D1 and down-regulated p53 and p21. Salidroside was presumed to inhibit growth and migration through down-regulation of miR-891b, leading to inactivation of PI3K/AKT/mTOR and NF-κB signaling pathways, but the direct molecular targets await clarification (Li H. et al., 2019).

**Paeonol** is a simple phenol found in peonies (*Paeonia* spp.) and other plants, that is used in traditional Chinese medicine (TCM). In a study on chondrosarcoma cells, Horng et al. (2014) showed that although paeonol did not decrease cell viability nor induce apoptosis, it exhibited anti-proliferative effects as evidenced by reduced protein-kinase C delta and c-Src phosphorylation activity. MiR-141 levels were elevated in treated cells while its suppression decreased the effects of paeonol. However, no mechanistic links were revealed between miR-141 and the protein-kinase C delta and c-Src pathways.

**Shikonin** is a naphthoquinone found in the purple gromwell plant (*Lithospermum erythrorhizon* Siebold and Zucc.), whose dried roots are used in TCM. Shikonin was found to reduce retinoblastoma cell proliferation by up-regulating miR-34a and miR-202, both well-known anti-oncogenic miRNAs. There is evidence for the inhibited proliferation being connected with miR-34a and miR-202 repressing the oncogene protein MYCN (Su et al., 2018). The inhibitory relationship between MYCN and miR-34a has been evaluated in lung cancer, where miR-34a increased chemosensitivity to cisplatin via inhibition of MYCN (Song et al., 2017).

### Flavonoids

Flavonoids are a wide family of phenolic compounds found in almost all plant organs including leaves, fruits or flowers. In addition to their role in the response against diverse biotic and abiotic stress factors, they are often responsible for the aroma and pigmentation of flowers and fruits (Erdman et al., 2007; Panche et al., 2016). This is to attract pollinators (in flowers) or to avoid being eaten by insects (in leaves). In terms of plant metabolism, these compounds have been described as hybrids, stemming from a combination of the phenylpropanoid and the polyketide pathways. For the synthesis of phenolic compounds, this is considered to be the most productive metabolic route in plants, with more than 8,000 compounds produced by different plant species being counted as belonging to the flavonoid family (Quideau et al., 2011). Flavonoids discussed in this review belong to several major flavonoid subclasses such as the flavones, flavanones, isoflavones, flavanols and chalcones.

In the case of flavones, there are multiple active chemicals that have been tested for anti-cancer properties. Among them, apigenin and luteolin are the most thoroughly studied.

**Apigenin** is a common flavone found in a range of plants, including *Matricaria* spp. (commonly known as chamomile), that displays important anti-tumor properties (Salehi et al., 2019). A study by Gao et al. (2018) found miR-520b, which physiologically acts as a tumor suppressor, to be up-regulated in apigenin treated hepatocellular carcinoma (HCC) cells**.** In turn, miR-520b targets ATG7, regulating autophagy and, in the cited study, leads to increased chemo-sensitivity to doxorubicin treatment. Apigenin treatment also showed increased miR-16 levels in glioma cell lines, which subsequently targeted and decreased BCL2 expression and down-regulated the NF-kB/MMP9 pathway (Chen X. J. et al., 2016).

As for **luteolin**, plants rich in this natural compound have been used in TCM for the treatment of hypertension, inflammatory skin diseases or cancer (Lin et al., 2008). *In vitro* and *in vivo* studies on hepatoma cells revealed that luteolin treatment promotes apoptosis and tumor suppression through up-regulation of miR-6809-5p which down-regulates Flotillin 1 by directly binding to the 3′UTR of Flotillin-1 gene mRNA (Yang et al., 2019a). Flotillin 1 is a membrane receptor protein that is considered to promote metastasis and tumor invasion by influencing the Erk1/2, p38, JNK and NF-κB/p65 signaling pathways (Lin C. et al., 2011; Cao et al., 2016).

In pancreatic cancer cells, luteolin treatment is associated with down-regulation of miR-301-3p, which directly targets caspase-8, an initiator of the extrinsic apoptosis pathway. Also, luteolin treatment and knockdown of miR-301-3p sensitized pancreatic cancer cells to the tumor necrosis factor (TNF)-related apoptosis-inducing ligand (TRAIL) (Moeng et al., 2020). This is a potent apoptosis inducer of the TNF cytokine superfamily, which was also tested as a recombinant anti-cancer agent, but with reported high resistance rates after repeated treatments (Wong et al., 2019).

Mir-34a, which has a well-known tumor suppressor role, has been shown to be epigenetically down-regulated by aberrant CpG methylation of its promoter region in multiple cancers, including gastric cancer (Lodygin et al., 2008). In a study on gastric cancer cell lines, the reduced expression of miR-34a was reversed by treatment with luteolin. Mir-34a was then shown to target and reduce expression of the BCL-2 protein, leading to apoptosis induction (Wu et al., 2015a).

Moreover, pleiotrophin (PTN), a growth factor that is highly expressed in several cancers associated with poor prognosis (Jee et al., 2016; Ma et al., 2017), has been found to be modulated by luteolin in colorectal cancer (CRC) cells through the up-regulation of miR-384, which binds to PTN 3′-UTR (Yao et al., 2019).

In human glioma cells, treatment with luteolin increased miR-124-3p expression, activated apoptosis through MAPK and the death receptor pathway. It also promoted autophagosome initiation following an increased ratio of LC3B II to LC3B I and decreased p62 levels. Although no mechanistic target was identified, the authors suggest PIM1 as a potential miR-124-3p target, an important protein for cell proliferation and apoptosis (Brasó-Maristany et al., 2016; Tursynbay et al., 2016; You et al., 2019).

Luteolin also showed synergistic effects with **silibinin**, a flavonolignan found in milk thistle seeds (*Silybum marianum* (L.) Gaertn.). In glioblastoma (GBM) cells, this specific co-treatment produced better results than conventional chemotherapy [bis-chloroethyl nitrosourea or temozolomide (TMZ)]. Co-treatment with these two flavonoid compounds increased apoptosis and reduced autophagy. This is owed to luteolin’s capacity of inhibiting PKCα and up-regulating mTOR and p62, along with silibinin’s ability to inhibit inducible nitric oxide synthase (iNOS). Their anti-oncogenic activity, regardless of p53 GBM status, is mediated by miR-7-1-3p overexpression. Mechanistically they showed that, miR-7-1-3p decreases *in vivo* the expression of XIAP, a potent anti-apoptotic protein (Chakrabarti and Ray, 2016).

**Baicalein** is a flavone found in the roots of plants such as the Baikal skullcap (*Scutellaria baicalensis* Georgi). Zhang et al. treated osteosarcoma cell lines with baicalein and noted increased miR-183 expression. The authors showed that this miRNA directly modulated the expression of Ezrin, an intracellular protein which connects the cell wall via the actin cytoskeleton. By decreasing Ezrin, baicalein treatment in this study led to decreased proliferation, invasion and migration (Zhang J. et al., 2018). These findings are additionally supported by another study of Zhang et al. (2014) who showed that Ezrin up-regulates N-cadherin levels and ERK pathway activity to promote osteosarcoma invasion. This mechanism has also been highlighted in other studies on different osteosarcoma and gastric cancer cells respectively (Zhu et al., 2012; Cao et al., 2014).

**Naringin** is the glycosidic form of the flavanone naringenin and can be found in citrus fruits. This compound was used by Tan et al. in human chondrosarcoma cells lines. The authors observed an up-regulation of miR-126, which was shown to regulate the expression of vascular cell adhesion molecule-1 (VCAM-1), a protein involved in cell motility, which probably accounted for the observed repression of migration and proliferation in this study (Tan et al., 2014).

**Formononetin,** an isoflavone derived from red clover (*Trifolium pratense* L.), has been investigated by Hu. et al., who used it to treat osteosarcoma cell lines and tumor bearing mice (Hu and Xiao, 2015). This study noted decreased expression levels of miR-375 and an increased Bax/Bcl-2 ratio, which probably accounts for the observed apoptosis induction *in vitro* and for the tumor mass shrinkage *in vivo*. Notably, formononetin also acts as a phytoestrogen and has been shown to act on the estrogen receptor-beta (ERβ) with anti-tumor effects on ER positive breast cancer cells (Chen et al., 2013). ER β is known to have anti-tumor effects in various tumors including osteosarcoma (Yang et al., 2017; Yang Z. M. et al., 2019). In breast cancer cell lines, miR-375 is up-regulated and inhibition resulted in decreased ERα signaling (de Souza Rocha Simonini et al., 2010).

The flavanols **epigallocatechin gallate (EGCG)** and **(−)-epicatechin gallate (ECG)** are important antioxidants from green tea and, as catechins, chemical precursors of condensed tannins (proanthocyanidins) (Quideau et al., 2011). In a study on osteosarcoma cell lines by Liangdong et al., EGCG increased apoptosis by cell cycle arrest in the G1 phase (Jiang L. et al., 2014). Arrest in the G1 phase was also observed in prostate cancer studies, without involvement of p53 (Gupta et al., 2003), while in leukemic cells the apoptotic effects were mediated through p53, p21 and Bax (which showed increased expression) and down-regulation of Bcl-2-α (Harakeh et al., 2008). Jiang et al. co-treated osteosarcoma cells with miR-126 and EGCG and showed that, in contrast to aforementioned findings, miR-126 did not interact with the mTOR pathway, without inducing the G1 phase arrest of the cell cycle. However, miR-126 enhanced the anti-tumoral effect of EGCG. Mechanistically, it remains unclear if EGCG can act as a miR-126 inducer (Jiang L. et al., 2014). However, studies on gastric cancer cell lines (Liu et al., 2014) and endothelial cell lines (Sui et al., 2014) showed that miR-126 regulates PLK2, PI3KR2, Crk, PI3K and Akt, respectively, and these pathways may be dysregulated in osteosarcoma cells as well.

In neuroblastoma cell lines, treatment with EGCG and ECG increased several tumor-suppressive miRNAs such as miR-7-1, miR-34a and miR-99a and decreased a number of oncogenic miRNAs such as miR-92, miR-93 and miR-106b. Overexpression of miR-7-1 followed by co-treatment with EGCG and fenretinide, a synthetic retinol derivate, increased apoptosis by promoting activation of the caspase-3 and calpain pathways. In contrast, miR-93 overexpression reduced the efficacy of fenretinide-EGCG co-treatment, promoted proliferation, with decreased caspase-8, caspase-3, tBid and calpain levels and increased expression of the apoptosis inhibitor survivin (Chakrabarti et al., 2012; Chakrabarti et al., 2013). In nasopharyngeal carcinoma cells, EGCG modulated miR-296 and decreased cancer cell migration. This effect was associated with down-regulation of signal transducer and activator of transcription 3 (STAT3) activation mediated by miR-296 (Lin et al., 2020).

**Xanthohumol** is a chalcone found in hops (*Humulus lupulus* L.). Ho et al. treated a U87-MG GBM cell line with xanthohumol and observed reduced invasiveness induced by the down-regulation of stromal interaction molecule 1 (STIM1). MiR-4725-3p was up-regulated in treated cells and it was shown that the effects of xanthohumol were mediated via miR-4725-3p, which binds directly to the 3′-UTR of STIM1 (Ho et al., 2018). STIM1 is an important protein in cellular calcium metabolism, and inhibition of STIM1 has favorable effects on proliferation and apoptosis (Liu et al., 2011). Furthermore, Chen et al. also treated glioblastoma cells with xanthohumol and noted apoptosis induction, as well as mitochondrial dysfunction and generation of radical oxygen species. The group demonstrated that xanthohumol up-regulated miR-204-3p via ERK/c-Fos pathway and went on to hypothesize IGFBP2 (insulin-like growth factor binding protein 2) as a potential target of this miRNA. IGFBP2 is implicated in the regulation of proliferation by modulation of AKT/Bcl-2, and its down-regulation through miR-204-3p may account for the observed cytotoxicity (Chen P. H. et al., 2016).

### Curcuminoids

Curcuminoids are phenolic compounds containing two benzene rings and are derived from the name-giving compound curcumin, featuring some variations in functional groups. **Curcumin** has been isolated from the turmeric plant (*Curcuma longa* L.) and is responsible for the distinctive bright yellow color of turmeric. Plants accumulate this insecticidal and fungicidal compound in the storage roots and other organs to protect them from being eaten or infected, respectively (Kim et al., 2003). A number of studies on different cancer cell lines reported curcumin down-regulating miR-21 levels with pro-apoptotic and anti-proliferative effects. In a diffuse large B-cell lymphoma cell line Liu et al. (Liu K. et al. (2017)) revealed VHL (Von Hippel-Landau), a tumor suppressor gene, as a target for miR-21. They then verified the involvement of curcumin by reversing all the salutary effects of this natural compound either through miR-21 overexpression or VHL siRNA transfection. In CRC, Mudduluru et al. (2011) showed that treatment of two CRC cell lines with curcumin down-regulated the miR-21 expression via regulation of activation protein 1 (AP-1) transcription factor. Subsequently, a known miR-21 target in CRC (Asangani et al., 2008), programmed cell death protein 4 (PDCD4) was shown to be up-regulated. Induction of cell cycle arrest in G_2_/M, *in vitro* anti-proliferative effect*s* and *in vivo* anti-metastatic effects by curcumin, might be a consequence of PDCD4 regulation via miR-21. Additionally, PDCD4 locus is coding also for an antisense lncRNA and an intronic miRNA which could play an additional role in the regulatory pathway.

Studies on breast cancer (Wang X. et al., 2017; Esmatabadi et al., 2017) and glioma (Yeh et al., 2015) have also reported curcumin down-regulating miR-21 expression, but without proposing a specific mechanism by which it does so, or possible targets of miR-21. Working with a GBM model, Tan et al. (2018) designed a micelle loaded with curcumin and miR-21 antisense nucleotide and reported increased effects compared to both curcumin treatment and miR-21 antisense nucleotide treatment alone. This was found both *in vitro* and *in vivo*, as evidenced by increased apoptosis and lower tumor volume, respectively. Tan et al. did not test for any miR-21 targets in this experiment, but showed elevated levels of phosphatase and tensin homolog (PTEN) and PDCD4, two known targets of miR-21 in other cancers (Cirino et al., 1991; Asangani et al., 2008).

In retinoblastoma cell lines, curcumin up-regulated miR-99a and reduced JAK/STAT activity in a miR-99a dependent manner, although the precise target of miR-99a was not identified (Li Y. et al., 2018). Sreenivasan et al. also treated retinoblastoma cells with curcumin and found that miR-22 was up-regulated and, upon subsequent transfection, cell migration was inhibited (Sreenivasan et al., 2012). This study, along with Patel et al. (2011) further showed that miR-22 negatively regulates Erbb3 which is up-regulated in various neoplasms including retinoblastoma (Chakraborty et al., 2007). Yu et al. (2015) showed that curcumin up-regulates miR-138 and down-regulates miR-186 in osteosarcoma cells. Moreover, they revealed Smad4, NF-kB p65 and Cyclin D3 as targets of miR-138 and demonstrated that curcumin inhibits these genes in a miR-138 dependent manner. It is also worth mentioning that miR-22 was also up-regulated, but downstream exploration was not undertaken.

Curcumin was used by Yin et al. (2018), Tahmasebi Mirgani et al. (2014) and Wu et al. (2015b) on GBM cells. Yin revealed that miR-326 and curcumin had complementary effects, with curcumin increasing miR-326 expression and miR-326 augmenting curcumin's anti-tumor activity by increasing apoptosis, inhibiting proliferation and migration (Yin et al., 2018). Furthermore, an inhibition of the SHH/GLI1 signaling pathway was observed, a result also described by other studies employing curcumin (Du et al., 2013) or miR-326 (Jiang Z. et al., 2014). In addition, a combined therapy decreased tumor volume and increased survival in a glioma mouse model population. While no specific target of miR-326 was identified by Yin in this experiment, SMO was evidenced as a direct target of miR-326 in another experiment on glioma cells (Du et al., 2015). Mirgani et al. showed that curcumin increased miR-145 expression and subsequently down-regulated OCT4A, OCT4B1 and SOX-2 which are known targets of miR-145 (Chivukula and Mendell, 2009; Xu et al., 2009) and reduced cell viability by increasing apoptosis and cell cycle arrest (Tahmasebi Mirgani et al., 2014). Because GBM is known for resistance to therapy with temozolomide, a process mediated through NF-kB overexpression (Lavon et al., 2007), Wu et al. investigated whether a combination of curcumin and TMZ would improve GBM chemosensitivity. Combination therapy resulted in increased apoptosis compared to either TMZ or curcumin alone and in an up-regulation of miR-146a levels (Wu et al., 2015b). Inhibition of miR-146a, which has been shown to negatively regulate the NF-kB pathway (Crone et al., 2012; Sha et al., 2013), canceled curcumin’s enhancing effect on TMZ treatment.

### Stilbenoids

Stilbenoids, a family of non-flavonoid phenolic phytochemicals, mainly play a role in fungal and UV protection in plants (Akinwumi et al., 2018). In humans, they are known to modulate several signaling pathways involved in oxidative stress and inflammation, therefore these compounds could have applications in cardiovascular protection, insulin resistance, neurodegeneration and cancer prevention (Carter et al., 2014; Kosuru et al., 2016; Dvorakova and Landa, 2017). One of the richest sources of stilbenoids currently known is the genus *Vitis*, which includes the grapevine *Vitis vinifera* L*.*

**Resveratrol** and its derivatives are the most extensively studied compounds in this class (Rivière et al., 2012) and can commonly be found in fruits such as red grapes and raspberries. In plants, they act as phytoalexins that play a role in the defense against bacterial and fungal pathogens (Tian and Liu, 2020). Xiao et al. investigated the relationship between miR-139-5p and resveratrol in osteosarcoma cells and discovered that resveratrol induces apoptosis and up-regulates miR-139-5p, with a synergistic relationship existing between the two molecules. While miR-139-5p mimics augment the effect of resveratrol, this effect is diminished by miR-139 inhibition. It was shown that miR-139-5p elicits an inhibitory effect on NOTCH1 by directly binding to the 3′ UTR (Xiao et al., 2020).

Yang et al. also treated osteosarcoma cells with resveratrol at non-cytotoxic concentrations and revealed that proliferation and invasiveness were inhibited *in vitro* and *in vivo*. Resveratrol up-regulated miR-328 which in turn directly binds MMP-2 and reduced its expression (Yang et al., 2015). Additionally, in a different study it was further demonstrated by another research group that miR-328-3p directly targeted MMP-16 in osteosarcoma cells (Zhang M. et al., 2019).

In a GBM cell line, resveratrol was found to down-regulate miR-21, in connection with a subsequent decreased phosphorylation of the Inbitor of κB (IκB) and p50/p65 heterodimer. This has the effect of blocking their nuclear internalization and the inability to activate the NF-kB pathway leads to a reduction of cell viability (Li H. et al., 2013).

MiR-15a and miR-16–1 are two anti-oncogenes that inhibit Bcl-2 and are known to be down-regulated in leukemia (Cimmino et al., 2005; Acunzo and Croce, 2016). Resveratrol exercised anti-tumor effects in T-cell and B-cell ALL cells by down-regulating miR-196b and miR-1290 which are known to be elevated in ALL lines (Zhou et al., 2017). Down-regulation of these miRNAs resulted in up-regulation of IGFBP3 (insulin-like growth factor binding protein 3), which was revealed to be lowered in ALL patients compared to healthy controls. Both miRNAs were found to bind to the 3′-UTR of the IGFBP3 gene with inhibitory effects.

**Pterostilbene** is an analog of resveratrol found in almonds, grapes and berries from the genus *Vaccinium*, with blueberries as the most noteworthy source. Huynh et al. investigated the effect of pterostilbene in CD133 + GBM stem cells with highly expressed GRP78 (Glucose-regulated protein 78), which are known for their resistance to therapy. Upon treatment with pterostilbene, an increase in miR-205, as well as a decrease in GRP78, c-Myc, TCF-4, GSK3β and vimentin levels were discovered. These modifications translated into the disruption of tumor sphere formation and radiation sensitization. The authors hypothesized that miR-205 is responsible for this down-regulation, as overexpression of miR-205 in monotherapy yielded the same results and combination therapy had a more pronounced effect. The findings were also confirmed *in vivo*, on xenograft mice (Huynh et al., 2015).

### Tannins

Tannins are polyphenolic secondary metabolites that often have high molar masses (up to 20,000 D) and that are known for their ability to crosslink collagen chains, an effect that is used in the process of turning animal skins into leather (“tanning”). They are found in a wide range of higher plant families and are responsible for the astringent taste of many plants. Their biological role mainly consists in protecting the plant against bacterial or fungal infection or herbivory (acting as a repellent), therefore an increase in tannin production is found when plants respond to these particular situations (Khanbabaee and van Ree, 2001). Based on their structural characteristics, tannins can be divided into several major groups: gallotannins, ellagitannins (often collectively referred to as hydrolyzable tannins), condensed tannins and complex tannins. Among these, condensed tannins (also called proanthocyanidins), which are derived from the oligomerization or polymerization of flavanol units, have shown promising effects in cancer research.

Chakrabarti et al. (2016) treated GBM cells with **grape seed proanthocyanidins (GSP)** and with miR-30e. Both GSP and miR-30e in monotherapy decreased autophagy as evidenced by reduced levels of Beclin-1 and LC3 II and increased apoptosis by down-regulation of BIRC6 and AVEN, two important apoptosis inhibitors. The role of autophagy, which is a physiologic process implicated in the household of healthy cells, turns to pro-tumoral in the context of hypoxia and starvation, by recycling proteins which contribute to the tumoral survival and proliferation. In the case of GSP and miR-30e, co-treatment showed synergistic results but with no evidence of a direct induction of miR-30e by GSP.

### Related Compounds

This section presents two more compounds belonging to two phytochemical families (lignans and flavaglines) with precursors stemming from the phenylpropanoid metabolic network (Pan et al., 2013; El-Seedi et al., 2018). While these particular compounds contain aromatic rings lacking free hydroxyl groups, they are included here, since they are biogenetically related to plant phenolics discussed above.

Lignans are building blocks of the cell wall, but they can also inhibit germination of competitor plants and protect the plant through their antimicrobial activity (DellaGreca et al., 2013). One lignan that has been studied in cancer research is **schisandrin**, a compound extracted from the magnolia-vine (*Schisandra chinensis* (Turcz.) Baill.). Jiang et al. (2015) found schisandrin to have an inhibitory effect on the mTOR/MMP-9 pathway and to inhibit glioma metastasis. In another study by Jiang et al., in which glioma cell lines were treated with schisandrin, an increase in miR-125a-5p expression and a decrease in the expression of the lncRNA *HOTAIR* (*HOX transcript antisense RNA*) were observed. This modulation of miRNA and lncRNA was associated with a lowered mTOR protein expression. Upon transfection with a miR-125a-5p inhibitor, the effects of schisandrin were diminished, but no further mechanistic details were offered (Jiang Y. et al., 2017). However, a molecular mechanism was described by Tang et al., who identified CASP2 as a target of miR-125a-5p and found that knockdown of *HOTAIR* can induce cell apoptosis via CASP2/miR-125a-5p axis (Tang et al., 2016).

Another compound tested for its potential use in cancer therapies is **silvestrol**, a flavagline produced by trees of the genus *Aglaia*, which showed important anti-tumor effects in acute myeloid leukemia (AML) cell lines. After FLT3-ITD (FLT3 internal tandem duplication) and FLT3-wt cell lines were subjected to silvestrol treatment, a reduction of proliferation and colony forming capacity and an increase of apoptosis were obtained in both cell lines. FLT3-ITD protein and miR-155 levels were decreased by silvestrol. NF-kB protein levels were found to be also decreased, which could indicate that silvestrol down-regulates miR-155 through NF-kB (Alachkar et al., 2013).

## Terpenoids and Meroterpenoids

Terpenoids are a group of molecules synthesized by all kinds of organisms (animals, plants, fungi, bacteria etc.) through either the mevalonate (MVA) or the methylerythritol phosphate (MEP) pathways, assuming a wide range of metabolic functions. They are particularly diverse in plants, with higher plants possessing a great number of enzymes specialized in terpenoid synthesis. In plants, some terpenoids can be classified as primary metabolites, as they contribute to essential cell functions like photosynthetic activity or membrane stability, while others that are involved in responses to environmental factors are counted as secondary metabolites (Bergman et al., 2019; Tetali, 2019). The latter category serves many functions similar to those previously presented for phenolic compounds: defense against pathogens and herbivores, attraction of pollinators or of natural enemies of herbivores, response to light stress etc. In fact, terpenoids form the most diverse group of secondary metabolites known from plants. A large number of terpenoids are used by humans for various reasons, including medical (e.g., as anti-malarial drugs or chemotherapy medication) or cosmetic purposes and in the food industry (Tetali, 2019).

Interestingly, in *Arabidopsis thaliana* (L.) Heynh., proper functioning of plant miRNAs in post-transcriptional gene silencing was shown to be dependent on terpenoid biosynthesis. As it has been suggested, this might be due to the need for a functional MVA pathway as a prerequisite for membrane association of Argonaute (AGO) proteins. As AGOs and, by extension, RNA-induced silencing complexes (RISC) are associated with membranes in both plants and animals, this might be an interesting example of phytochemicals influencing miRNA function through a common mechanism in both groups of organisms (Brodersen et al., 2012).

Terpenes represent the basic form of terpenoids. They are derived from isoprene precursors containing five carbon atoms and can be classified by the number of isoprene units they are composed of, e.g., hemiterpenes (1 unit–5 C), monoterpenes (2 units–10 C), diterpenes (4 units–20 C) up to polyterpenes that may consist of thousands to tens of thousands of isoprene units. The term terpenoids is used to designate a broader category which comprises terpenes and derived molecules containing additional functional groups. This section additionally includes terpenoid glycosides and molecules having at least a partial terpenoid structure (meroterpenoids), like terpenophenols or other prenylated compounds.

### Monoterpenoids, Diterpenoids and Triterpenoids

**α-pinene** is an isomer of pinene, a monoterpene found in the resin of coniferous plants. Xu Q. et al. (2018) and Yang J. B. et al. (2016) showed that α-pinene induced G2/M phase cell cycle arrest and inhibited miR-221 expression with downstream up-regulation of CDKN1B/P27 and down-regulation of CDKN1C/P57 in HCC cells. The mechanism through which miR-221 regulates CDKN1C/P57 and CDKN1B/P27 was not elucidated in this study, but another study revealed that CDKN1B/P27 is a direct target of miR-221 (Diaz-Moralli et al., 2013).

**Ailanthone** is a pentacyclic diterpene lactone produced by the tree of heaven (*Ailanthus altissima* (Mill.) Swingle). This compound was used to treat a number of cancer cell lines with proapoptotic and anti-proliferative effects and showed regulatory activity on a number of different miRNAs. In a study on AML cell lines (Zhang Y. et al., 2019), ailanthone up-regulated miR-449a with inhibitory effects on the Notch and PI3K/AKT pathways, both of which were demonstrated to be downstream targets of miR-449a (Liu X. et al., 2018; Cheng et al., 2018). Similar proapoptotic and anti-proliferative effects were observed by Gao W. et al. (2019) in breast cancer cell lines, where ailanthone up-regulated miR-148a and inhibited the AMPK and Wnt/β-catenin pathways in a miR-148a-dependent manner. For Hou et al. of course in a study on lung cancer cell lines, also showed that ailanthone blocked the PI3K/AKT pathway as well as the JAK/STAT3 pathway in a miR-195-dependent manner, miRNA levels being up-regulated upon treatment (Hou et al., 2019). Yang et al. revealed that miR-21 was down-regulated by ailanthone in a schwannoma cell line with downstream inhibition of the Ras/Raf/MEK/ERK and mTOR pathways. MiR-21 overexpression partly reversed ailanthone effects by increasing the expression of Ras, Raf, *p*-MEK, *p*-ERK, *p*-mTOR and p-p70S6K (Yang P. et al., 2018).

**Carnosic acid** is a diterpenoid found in rosemary (*Salvia rosmarinus* Spenn.) and sage (*Salvia officinalis* L.). Liu and colleagues induced G2/M phase cell cycle arrest and lowered proliferation in a chronic myeloid leukemia (CML) cell line through treatment with carnosic acid. MiR-708 was down-regulated, but no downstream pathway mechanisms were explored (Liu D. et al., 2018). It is worth mentioning that miR-708 has been reported as both an oncogenic (Li X. et al., 2013; Zhang Y. et al., 2017; Huang et al., 2018) and an anti-oncogenic (Baer et al., 2015; Wu et al., 2016; Monteleone and Lutz, 2020) miRNA in various other studies on leukemic and lung cancer cells. A study using rosemary extracts on colon and pancreatic cancer cells showed anti-cancer effects both *in vitro* and *in vivo* (colon cancer xenografts in treated nude mice showed reduced tumor size compared to untreated mice). In this study, the authors showed that carnosic acid down-regulates miR-15b, subsequently leading to overexpression of the *GCNT3* gene. However, a direct interaction between this miRNA and *GCNT3* was not identified (González-Vallinas et al., 2014).

**Triptolide** is a diterpenoid extracted from the Asian medicinal plant *Tripterygium wilfordii* Hook. f., which has been used in TCM against rheumatoid arthritis and psoriasis (Bao and Dai, 2011). This compound has shown anti-cancer effects in studies employing a variety of cancer cell lines.

In medulloblastoma cells treated with triptolide, miR-138 was increased and proliferation, viability and migration were inhibited. MiR-138 directly targets Cyclin Dependent kinase 6 (CDK6). Hence, a possible mechanistic explanation for the anti-cancer effect of triptolide is the inhibition of CDK6 via miR-138 (Zhang H. et al., 2018). Furthermore, triptolide also induced the apoptosis of osteosarcoma cells by inhibition of PTEN. MiR-181a was up-regulated by triptolide and directly inhibited PTEN mRNA translation (Jiang C. et al., 2017).

In CRC, treatment with triptolide down-regulated miR-191 and was associated with decreased activation of Wnt/β-catenin and NFkB pathways. Treated cells showed increased apoptosis and reduced migration capabilities (Qi and Li, 2019). Nephroblastoma cells treated with triptolide showed a decrease in KLF-4 activity (an activator of p53) and down-regulation of PI3K/AKT and ERK pathways. KLF-4 activity was modulated by miR-193b-3p (Hang et al., 2019).

In ALL cell lines, 21 different miRNAs were modulated by triptolide. MiR-138-2^*^ was the most significantly up-regulated and miR-16–1 was the most significantly down-regulated, supposedly accounting for the anti-proliferative effects of triptolide noted in this study (Meng et al., 2011). Furthermore, breast cancer cells showed decreased invasion and migration capacity after exposure to triptolide. Triptolide up-regulates miR-146a, which mechanistically down-regulates the expression of Ras-related C3 botulinum toxin substrate 1 (RAC1), an important cellular switch which is altered in cancerous states (Liu Q. et al., 2019).

**Betulinic acid** is a triterpenoid found in the bark of several tree species, most notably that of the white birch (*Betula pubescens* Ehrh.), where it functions as a phytoalexin. This compound was used to treat breast cancer cell lines by (Mertens-Talcott et al., 2013) and (Liu et al., 2012). Both groups showed that betulinic acid displays anti-cancer activity, as reflected in lower cell survival, proliferation and increased apoptosis. Betulinic acid down-regulated the transcription factors SP1, SP3 and SP4 via miR-27a down-regulation, and the subsequent up-regulation of its target ZBTB10. Furthermore, Liu et al. demonstrated that betulinic acid effect on miR-27a-ZBTB10-Sp axis was CB1 and CB2 cannabinoid receptors dependent.

### Carotenoids

Carotenoids are lipid-soluble tetraterpenoid pigments fulfilling important functions in plants, such as facilitating photosynthesis by harvesting light energy (photons) as an antenna pigment, protecting against high light-induced damage or signalizing fruit ripeness. They are often responsible for yellow, orange or red coloration in different parts of the plant (Ngamwonglumlert et al., 2019).

One of the best-known carotenoids, **β-carotene**, has been tested in colon cancer stem cells, where it reduced sphere formation and proliferation and it was shown to act as an epigenetic regulator by down-regulating various miRNAs, such as miR-1260b and miR-296-3p. Mechanistically, these dysregulated miRNAs stimulated the acetylation process, reflected by an increase in histones H3 and H4, as well as the reduced expression levels of the DNMT3A mRNA, resulting in DNA hypomethylation. Both these noted effects are counteracting epigenetic modifications observed in cancer cells (Kim et al., 2019).

In human diets, β-carotene functions as provitamin A consisting of two retinyl groups and is transformed in the digestive system into retinol, a form of vitamin A. Due to some carotenoids (including β-carotene) being sources of vitamin A, we will proceed to discuss the effects of a synthetic isoform of vitamin A called **all-*trans* retinoic acid (ATRA).** While it is not a natural compound itself, ATRA is synthesized from β-carotene (oxidative cleavage of carotenoids catalyzed by enzyme models and beta-carotene 15,15′-monooxygenase) and, therefore, it is a very suitable example for demonstrating the value of developing synthetic substances inspired by natural compounds in an attempt to optimize their known beneficial effects.

In a recent systematic review by Lima et al. (2019), the effects of ATRA on nine different *in vitro* cancer types was summarized. In this review, miRNA expression profiles, as well as phenotypical changes after ATRA treatment are well described for the nine cancer forms. However, for most of the covered studies, the mechanistic description of miRNA downstream pathways is missing.

**FIGURE 2:**
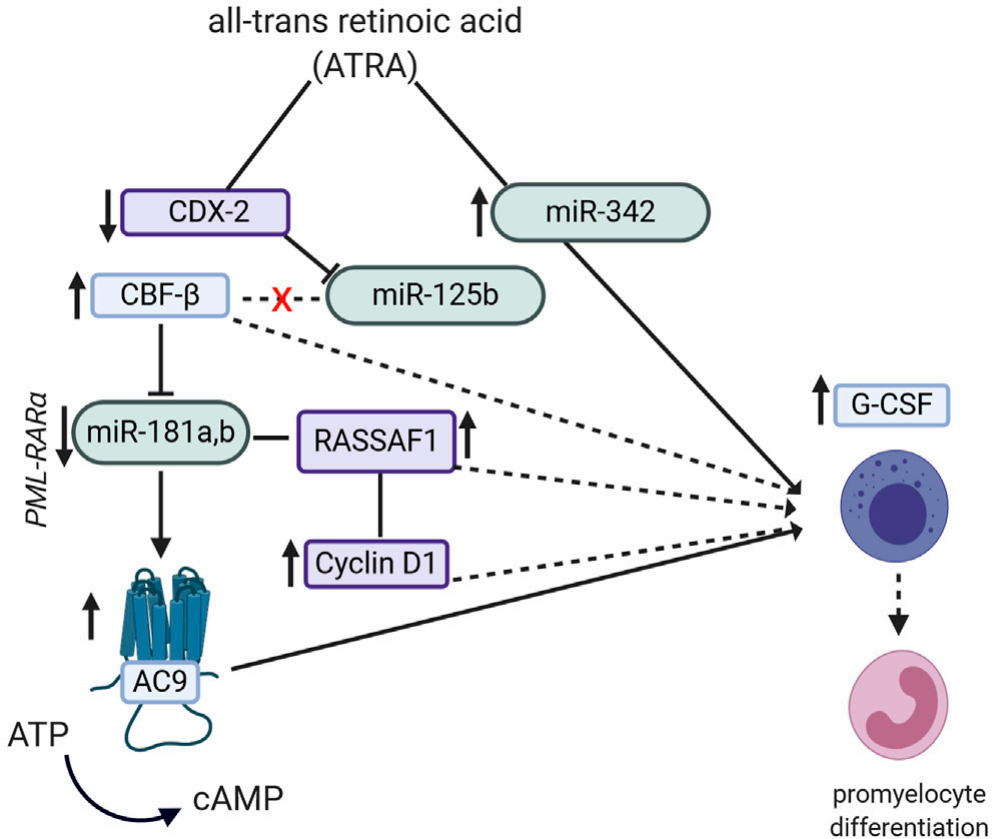
Potential pathways by which the semi-synthetic all-trans retinoic acid (ATRA) compound can lead to promyelocyte differentiation in promyelocytic leukemia (PML). ATRA induces miR-342 up-regulation, which then leads to increased expression of G-CSF mRNA. ATRA decreases expression of homebox protein CDX2 and miR-125b as well as increases the expression of core binding factor β (CBFβ). CBFβ can also modulate expression levels of miR-181a and b, which in turn modulates RASSAF1/Cyclin D1 expression, finally contributing to promyelocytic differentiation.

Nevertheless, the ATRA-induced promyelocytic leukemia (PML) differentiation through miRNA is covered in different studies, numerous mechanistic explanations being available. In one study (De Marchis et al., 2009), ATRA achieved an up-regulation of miR-342, while at the same time increased expression levels of granulocyte colony-stimulating factor receptor (G-CSFr) mRNA levels were noted, which in turn, were linked to promyelocytic differentiation. Likewise, in another study on AML lines, the research group (Lin K. Y. et al., 2011) showed that ATRA could down-regulate the expression of the homebox protein CDX2, stopping it from binding to the promoter region of the miR-125b gene, hence down-regulating its expression. The down-regulated miR-125b then stops binding to the core binding factor β (CBFβ) gene, allowing it to be expressed, which also accounted for the granulocytic differentiation. Interestingly, in a different study (Zhuang et al., 2014), it was shown that CBFβ down-regulates the expression of miR-181a which, in turn, regulates the adenylate cyclase 9 (AC9) levels. The latter is an enzyme which catalyzes the conversion of ATP to cAMP, which is required in the differentiation process of the promyelocytic forms. These findings are confirmed by a different study which showed that the promyelocytic differentiation is miR-181a,b-dependent. In PML, the miR-181 cluster is pathologically up-regulated because of the PML/RARα oncogene. Upon ATRA treatment, levels of the miR-181a,b are down-regulated. In this study, these miRNAs were shown to bind to the 3′UTR gene of RASSF1A, regulating its expression. While down-regulation of RASSFA1 halted the ATRA-induced differentiation by influencing the cyclin D1 pathway, its overexpression led to apoptosis (Bräuer-Hartmann et al., 2015). In non-small-cell lung cancer (NSCLC) lines, ATRA induced the up-regulation of miR-512-5p, which in turn may be responsible for the observed p21 mRNA and protein down-regulation, associated with apoptosis and lesser glucose uptake by cancer cells (Chu et al., 2016). These finding are represented in Figure 2.

### Saponins

Saponins are glycosylated triterpenoids with a wide distribution in plants, where they are mainly associated with defense responses against herbivores (acting as antifeedants) and pathogens (Sawai and Saito, 2011). Most of them are amphipathic, being intercalated in the lipid bilayers where, among other functions, they influence membrane permeability (Moses et al., 2014). They have also been used for ages in traditional medicine and most compounds used in cancer-related studies featuring ncRNAs generally originate from TCM. The majority of the studies are conducted on different cancer cell lines, while others also focus on *in vivo* models of tumor-bearing mice.

Some of the most extensively studied saponins are derived from the ginseng genus *Panax*, most commonly from the species *Panax ginseng* C.A.Mey and *Panax notoginseng* (Burkill) F.H.Chen. Among these compounds, the most thoroughly investigated is **Ginsenoside Rh2**, which has been shown to have anti-proliferative and/or apoptosis-inducing properties. In glioma cell lines, Rh2 was shown to regulate expression of different miRNAs. Especially an up-regulation of miR-128 was noted, which was then demonstrated to target the promoter region of E2F3a, reducing its expression. E2Fa is a transcription factor implicated in regulating expression of key components of the cell cycle, which explains the noted anti-proliferative effects. Moreover, in the same study, increasing caspase-3 levels were noted along dose- and time-dependent induction of apoptosis through Rh2 (Wu et al., 2011). Likewise, in prostate cancer cell lines, miR-4295 was shown to be suppressed by Rh2, not being able to bind to the 3′UTR region of CDKN1. This resulted in up-regulation of p21, with known implications in cell cycle regulation, accounting for the observed anti-proliferative effects (Gao and Zheng, 2018).

In a study on both hepatitis B-induced HCC cell lines as well as on tumor bearing mice, Rh2 also showed anti-proliferative potential by up-regulation of miR-491, which was shown to target the epidermal growth factor receptor (EGFR) and by reducing its expression to act anti-proliferative (Chen and Qiu, 2015). In one study on NSCLC lines, Rh2 was proposed as an anti-metastatic agent. It was shown to up-regulate miR-491, which in turn might reverse the hypoxia (HIF-1α)-induced up-regulation of MMP-9, contributing to the anti-migratory effect noticed in the cell lines (Chen Y. et al., 2019).

In a study on adriamycin- and docetaxel-resistant breast cancer cell lines, Wen et al. showed anti-proliferative effects after treatment with Rh2. The authors hypothesized that Rh2 could reverse chemoresistance by regulating the expression miR-222, miR-34a and miR-29a, which in turn could target effectors implicated in chemoresistance reversal. However, no targets of specified miRNAs were analyzed in this study, only an unspecific increase in BAX expression levels was noted (Wen et al., 2015).

Ginsenoside Rh2 was proposed to induce apoptosis and to prolong survival in mice with AML and the miR-21/Bcl-2 pathway (Wang and Wang, 2015). In retinoblastoma cell lines it also showed anti-proliferative and apoptosis inducing properties via miR-638 via PI3K/AKT/mTOR pathway (Li M. et al., 2019).

A related compound, **ginsenoside Rg3** was proposed to influence the cancer cell metabolism in two studies on ovarian cell lines by reversing the Warburg effect. In one of the studies, Rg3 decreased expression of several lncRNAs including *lncH19*, which was shown to target miR-324-5p. The increased miR-324-5p levels inhibited pyruvate kinase isozyme 2 (PKM2), which accounted for the noted metabolic change (Zheng et al., 2018). In a similar follow-up study, the same research group noted that Rg3 inhibited the expression of DNA methyltransferase 3A (DNMT3A), which, in turn, reduced methylation of many genes, among these also the promoter region of miR-532-3p leading to the up-regulation of this miRNA. Further on, this miRNA was shown to target hexokinase 2 (HK2), having the same consequence, Warburg effect reversal (Zhou et al., 2018).

Another similar compound, **ginsenoside Rg2** was tested also on oral squamous cell cancer (OSCC) cell lines where it showed anti-proliferative and anti-metastatic effects by down-regulating miR-221. Low levels of miR-221 up-regulate the metalloproteinase inhibitor 3 (TIMP3), which in turn acts on the PI3K/AKT and MAPK/ERK pathways. The findings were also confirmed on tumor bearing mice (Cheng and Xing, 2019).

There is also a third type of ginsenoside that has been investigated, **ginsenoside Rd.** This compound showed anti-metastatic properties *in vitro* and *in vivo* in breast cancer via down-regulation of miR-18a, which directly targets Smad2, known to be implicated in cell migration (Wang P. et al., 2016).

One study showed that a mixture of ginsenosides could also influence angiogenesis in a tissue specific manner via miR-18a, reducing neo-vascularization in tumor tissue, while stimulating it in cardiac tissue of the same tumor-bearing mice (Yang Q. et al., 2014). Another study focused on the **P. notoginseng** extract (a mixture of multiple ginsenosides), showed anti-proliferative effects *in vitro* and *in vivo* also via miR-222, by targeting and up-regulating p27 and PTEN tumor suppressors, which in turn modulated the expression of several other oncogenes or tumor suppressor genes in lung cancer cell lines. Results were confirmed *in vivo* on inoculated mice (Yang Q. et al., 2016).

Of course, saponins known to have miRNA-dependent anti-cancer effects are found in other plants as well, beside ginseng species. For instance, **polyphyllin VI**, extracted from the Asian plant species *Paris polyphylla* Sm., has been tested in murine and human breast cancer lines and was shown to decrease miR-18a expression, up-regulating the Receptor expressed in lymphoid tissue (RELT)-like 2 (Rell2), which was linked to the observed anti-metastatic effects (Wang et al., 2019)**.**

**Astragaloside IV** is a saponin extracted from the root of the Mongolian milkvetch (*Astragalus membranaceus* (Fisch.) Bunge). This compound was shown to improve tumor microenvironment (TME) disbalance and affect EMT and migratory potential in fibroblast derived from gastric cancers (in opposition to fibroblasts derived from non-cancerous gastric tissue) via miR-214 and miR-301. The two miRNAs subsequently modulate M-CSF and TIMP2 levels, which, in turn, prevented the expression of the pro-oncogenic factors SRY (sex determining region Y)-box 2 (SOX2) and NANOG (Wang Z. F. et al., 2017). In CRC, it also affected EMT through miR-134/CREB1, while it also increased chemosensitivity through the same mechanism (Ye et al., 2017). Cell proliferation was also reduced in CRC via miR-29c and B7-H3 protein (Wang et al., 2018).

In another study in HCC, astragaloside IV together with curcumin, reduced angiogenesis via miR-122 and miR-212 *in vivo*, by influencing expression of several TME regulatory molecules (FGF2, MMP2, VEGF, HGF, TF, FVII). The data was also confirmed by microscopical vessel count (Zhang S. et al., 2017).

There are also several saponins with anti-proliferative and anti-migratory properties on NSCLC lines. For instance, NSCLC cell lines treated with **Tubeimoside I,** have been shown to have increased miR-126-5p expression. In turn, miR-126-5p targeted VEGF-A and VEGFR-2, leading to reduced angiogenesis and reduced metastatic potential (Shi et al., 2018). Still in NSCLC cell lines, ***Cyclamen pseudibericum*** Hildebr. **extract** up-regulated miR-200c which, in turn, targeted the transcriptional repressor Zinc finger E-box-binding homeobox 1 (ZEB1), resulting in decreased expression of E-cadherin, N-cadherin and vimentin, reflecting the EMT-inhibitory properties of this extract (Karagur et al., 2018).

Other saponins were found to have anti-cancer effects in studies addressing a variety of cancer types. In hepatoma cell lines, a saponin extacted from the plant species *Ornithogalum saundersiae* Baker called **OSW-1** (alternatively known as orsaponin), in combination with doxorubicin influenced the expression of multiple miRNAs (especially miR-142-3p), which are known to be implicated in tumor metabolism (Jin et al., 2013). In gastric cancer, another anti-proliferative saponin was **Platycodin D**, which regulated miR-34a/surviving levels *in vitro* and also *in vivo* (Peng et al., 2019). **D Rhamnose β-hederin** from the vine *Clematis ganpiniana* (H.Lév. and Vaniot) Tamura reduced breast cancer tumor cell exosome production, exosomes which either carry genetic cargo coding for Docetaxel chemoresistance (Chen et al., 2018b) or simply pro-metastatic miRNAs, like miR-130 and miR-425 (Chen et al., 2018a). Finally, in breast cancer cell lines, **Timosaponin A-III,** extracted from the plant species *Anemarrhena asphodeloides* Bunge, has been shown to induce the expression of miR-200c and miR-141, both of which may target c-Myc. This might lead to the down-regulation of the BMI1, a protein associated a prooncogenic phenotype in several malignancies (Gergely et al., 2018).

### Polyprenylated Acylphloroglucinols

Polyprenylated acylphloroglucinols are a peculiar class of natural compounds that contain multiple isoprenyl or geranyl chains stemming from the MVA/MEP biosynthetic pathways coupled to an acylphloroglucinol core moiety derived from the polyketide pathway. These meroterpenoid compounds are very diverse in terms of structure and bioactivity. A subgroup with less complex structures, monocyclic polyprenylated acylphloroglucinols (MPAPs) is known from plant families such as Clusiaceae (Guttiferae), Myrtaceae and Cannabaceae (Ciochina and Grossman, 2006), while their more complex derivatives, the polycyclic polyprenylated acylphloroglucinols (PPAPs) have been isolated almost exclusively from the genera *Hypericum* and *Garcinia* of the family Clusiaceae. Only two exceptions from the families Rutaceae and Simaroubaceae have recently been described (Yang X. W. et al., 2018).

**Garcinol** is a PPAP possessing a dihydroxyphenyl moiety extracted from the fruit of the kokum tree (*Garcinia indica* (Thouars) Choisy). Kokum fruits are widely used in Indian cuisine as a souring agent and for the preparation of refreshing drinks (Padhye et al., 2009). In pancreatic cancer cell cultures (PANC-1SP cells), garcinol treatment effectively increased the expression of different anti-oncogenic miRNAs: miR-200c, miR-29b, miR-101 and miR-181. Only the activity of miR-200c was investigated in more detail. MiR-200c shows affinity for the 3′UTR of the *NOTCH1* mRNA, with its subsequent down-regulation leading to a reversal of cancer stemness (including reduction of self-renewal ability) and metastasis inhibition (Huang et al., 2017). Similarly, a study by Parasramka et al. found the modulation of several miRNAs by garcinol to be the mechanism behind the reduction in tumor sphere formation. Additionally, this regulation of miRNA expression was suggested to be involved in the attenuation of the drug resistance phenotype when garcinol was used alongside gemcitabine. Garcinol and gemcitabine co-treatment modulates several miRNAs involved in canonical cancer signaling pathways such as miR-21, miR-196a, miR-495, miR-605, miR-638, miR-453, with gemcitabine alone modulating only miR-605. Meanwhile, garcinol itself was shown to up-regulate miR-453, miR-128, miR-1280 and miR-720 expression and down-regulate miR-21, miR-495, miR-494, miR-1977 (Parasramka et al., 2013).

The ability of garcinol to increase the sensitivity of cancer cells to other drugs was also described in a study on mesenchymal NSCLC cell lines (Farhan et al., 2019). Here, garcinol treatment resulted in drug-sensitization to cisplatin and erlotinib as well as in EMT inhibition. EMT is regulated by several important miRNAs such as miR-200b, miR-205, miR-218 and let-7c, all of which were up-regulated by garcinol in these cell lines, with miR-200b and let-7c levels being the most strongly influenced.

Furthermore, in breast cancer cell cultures and xenograft mouse models, garcinol treatment facilitated the transition of EMT to MET phenotype. Several miRNAs were significantly up-regulated such as let-7 family miRNAs (let-7a, let-7e, let-7f) and members of the miR-200 family (miR-200b, and miR-200c). MiR-200b and mirR-200c up-regulation is associated with decreased activity of the NF-kB signaling pathway and provides a molecular framework for how garcinol lowers cancer cell invasiveness (Ahmad et al., 2012).

### Cannabinoids

Cannabinoids are a group of terpenophenols (meroterpenoids) with 21 carbon atoms naturally occurring in hemp (*Cannabis sativa* L.), known for their capacity to bind the cannabinoid receptors CB1 and CB2. This characteristic offers them psychoactive properties as well as the ability to modulate the immune system (Widelski et al., 2017). Two very well-known examples of natural cannabinoids are tetrahydrocannabinol (THC) and cannabidiol (CBD).

The anti-inflammatory effects of cannabinoids, particularly of **cannabidiol (CBD)** have been demonstrated in several studies on microglial cells exposed to CBD and lipopolysaccharide (LPS). MiR-146a, an important element of the Toll like receptor (TLR) signaling pathway (O'Neill et al., 2011), was up-regulated by LPS and down-regulated by CBD. In addition, pretreatment with CBD reduced miR-146a up-regulation after exposure to LPS (Juknat et al., 2019). A study on low-grade gliomas in a pediatric population revealed that the low expression of the CB1 receptor and high levels of hsa-miR-29b-3p are potential prognostic factors for involution or absence of progression after subtotal tumor resection (Sredni et al., 2016). These results show that further research into miR-29b-3p and exogenous and endogenous cannabinoids is needed.

## Alkaloids

Alkaloids are a class of natural compounds with structures containing at least one nitrogen atom. These compounds are immensely structurally diverse and are primarily connected to chemical defense mechanisms developed by plants against herbivores, pathogens or competing plants. Therefore, many alkaloids show significant levels of toxicity (Michael, 2007). Many are also used for medical purposes. Some alkaloids such as vincristine and vinblastine are already being used as chemotherapeutic agents in a number of cancers such as Hodgkin’s lymphoma, neuroblastoma and Wilms tumor (Moudi et al., 2013; Below and Das, 2020). Many other alkaloids have been studied in order to ascertain their effects either as direct anti-cancer agents or as adjuvants by increasing chemotherapeutic sensitivity (Mondal et al., 2019).

**Neferine** is an alkaloid found in the seeds of the sacred lotus (*Nelumbo nucifera* Gaertn.), which are used in TCM to obtain a herbal remedy known as Lotus Plumule or Plumula Nelumbinis. Liang et al. treated glioma cell lines with neferine and found increased apoptosis along with decreased cell proliferation and migration. Neferine down-regulated miR-10b which was found to inhibit PTEN/PI3K/AKT by targeting *PTEN* gene. Subsequently, PTEN expression–an important tumor-suppressive gene-was increased in U251 treated cells. p38-MAPK pathway was deactivated with lower p38 phosphorylation levels following neferine treatment via down-regulation of miR-10b (Liang et al., 2019). However, no further inquiries regarding the molecular target were formulated.

Liu Z. et al. (2019) also achieved anti-tumor effects with neferine on a breast cancer cell line through down-regulation of miR-374a. This miRNA was further shown to increase Fibroblast growth factor receptor 2 (FGFR-2) expression. Neferine inhibited the PI3K/AKT and MEK/ERK pathways through the modulation of miR-374a/FGFR-2 axis leading to reduced cell proliferation and migration and enhanced apoptosis. Neferine also inhibited the PI3K/AKT pathway in a gastrointestinal stromal tumor cell line by up-regulating miR-449a levels (Xue et al., 2019).

**Berbamine** is an alkaloid found in the Amur barberry plant (*Berberis amurensis* Rupr.). A synthetic derivative (BBMD3) of berbamine was used by Yang F. et al. (2014) to treat glioblastoma cell lines. Apoptosis was increased by caspase-3 and JNK-c JUN/AP-1 pathway activation as evidenced by increased cleavage of poly (ADP-ribose) polymerase and phosphorylation of c-Jun and c-Fos respectively. MiR-4284 was one of several up-regulated miRNAs, and its inhibition resulted in diminished apoptotic effects of BBMD3. In a study on melanoma cells, BBMD3 inhibited the STAT3 pathway (Nam et al., 2012), but these results were not confirmed by Yang et al. in their studies.

**Matrine**, oxymatrine (matrine oxide) and sophocarpine are alkaloids found in plants of the genus *Sophora* of the legume family (Fabaceae). Matrine induced apoptosis and cell cycle arrest in a number of different cancer cell lines. In breast cancer, matrine acted through the miR-21/PTEN/Akt pathway by down-regulating miR-21 to induce apoptosis and cell cycle arrest in the G1/S phase (Li et al., 2012). In a study on colon cancer cells, matrine, by up-regulating miR-22 which was found to target ERBB3 and MECOM mRNA. This way, matrine induced apoptosis and G0/G1 cell cycle arrest as well as down-regulation of the Wnt/β-catenin and MEK/ERK pathways (Liu J. et al., 2020). MiR-126 was also up-regulated in a NSCLC cell line treated with matrine and increased apoptosis and G0/G1 phase arrest were also observed (An et al., 2016). In melanoma cells, matrine also up-regulated PTEN and modulated the PTEN/Akt pathway to promote apoptosis, but by means of down-regulating miR-19b-3p which was also found to bind to PTEN as did miR-21 in breast cancer cells (Wei et al., 2018).

MiR-21 was also down-regulated by **sophocarpine** in a head and neck squamous cell carcinoma cell line by inhibition of Dicer processing and resulted in increased activity in the PTEN and p38/MAPK pathways. Beneficial effects were obtained both *in vitro* with increased apoptosis but also *in vivo* with growth inhibition in a mouse xenograft model (Li W. et al., 2017).

Anti-cancer effects were also described for **oxymatrine**. Li et al. (2015) found up-regulated levels of miR-29b with downstream inhibition of MMP-2 expression in ovarian cancer cells treated with oxymatrine. In another study on lung cancer cells, Zhou et al. (2019) also managed to lower proliferation both *in vitro* and *in vivo* by up-regulating miR-520 which showed inhibitory effects on VEGF expression after oxymatrine treatment.

Solanine and solasodine are toxic alkaloids found in some very common representatives of the nightshade family (Solanaceae), including tomatoes, eggplants and the fruits of potato plants. Zhang F. et al. (2016) and Wu et al. (2018) both reported that **solanine** up-regulated miR-138 with anti-proliferative and chemosensitizing effects in lung adenocarcinoma cells and esophageal cancer respectively. On lung adenocarcinoma cells, solanine was cytotoxic by itself and also improved chemosensitivity to cisplatin. Focal adhesion kinase (FAK) levels, a protein involved in cellular adhesion that was investigated as a potential therapeutic target for lung (Kawashima and Kurokawa, 1983) and breast (Baraka and Serhal, 1989; Zhang S. et al., 2017) cancer (among others), were down-regulated. FAK was revealed to be a direct target of miR-138 and it was shown that upon antisense transfection, solanine manifested no anti-cancer effects.

Regarding esophageal cancer, solanine improved chemosensitivity to 5-FU and cisplatin in a miR-138 dependent manner. Survivin, a protein which inhibits apoptosis and is highly expressed in tumor tissue (Sah et al., 2006), was down-regulated by miR-138 which directly bound to its mRNA. MiR-138 antisense transfection lifted the effects of solanine on survivin expression. In a study on prostate cancer cells, solanine up-regulated GAS5, a lncRNA, which was revealed to target miR-18a directly and decrease its expression with increased radiosensitivity as a result. GAS5 was overexpressed without solanine and, indeed, the same proapoptotic effects were seen, however no target of miR-18a was mentioned (Yang et al., 2019a).

The related compound **solasodine** down-regulated miR-21 in a lung cancer cell line and besides inhibiting the PI3K/Akt pathway it was shown to increase that expression of RECK (reversion-inducing cystein-rich protein with kazal motifs). RECK is known target of miR-21 and acts as metastasis suppressor (Shen et al., 2017).

**Capsaicin** is an alkaloid that is found in *Capsicum* peppers, within the Solanaceae family*.* By binding to capsaicin receptors in our tongues, it triggers a sensation of heat or pungency, which accounts for its wide use as a spice in global cuisine. CML cells treated with capsaicin showed decreased proliferation, with capsaicin down-regulating miR-520a-5p and, by a miR-520a-5p-dependent mechanism, also down-regulating the STAT3 pathway (Kaymaz et al., 2014). Acting as a reactive oxygen species (ROS), capsaicin damages DNA, which triggers cell apoptosis through the p53/miR-34 axis, as was show in a NSCLC cell line. It is worth mentioning that absence of p53 abolished the effects of capsaicin (Chakraborty et al., 2014). Interestingly, capsaicin also inhibited proliferation of prostate cancer cells, not through increasing apoptosis, rather through inducing cell cycle arrest in the G0/G1 phase. Capsaicin up-regulated miR-449a, which in turn dowregulated the androgen receptor (AR) (Zheng et al., 2015).

**Ligustrazine** is an alkaloid found in nattō, a traditional Japanese dish of fermented soybeans, in fermented cocoa beans and in *Ligusticum wallichii* Franch., a plant of the Apiaceae family used in TCM. A study (Xu D. et al., 2018) on medulloblastoma cell lines showed that ligustrazine promoted apoptosis and inhibited proliferation and invasion by inhibiting the mTOR and PI3K/Akt pathways. Ligustrazine also up-regulated miR-211 and although miR-211 antisense transfection reduced ligustrazine's inhibitory effects on the above-mentioned pathways, no direct target of miR-211 was evidenced.

**Sinomenine** is an alkaloid found in the roots of *Sinomenium acutum* (Thunb.) Rehder and E.H.Wilson, a climbing plant from East Asia used in TCM. Sinomenine showed proapoptotic effects in prostate cancer cells by inhibition of the PI3K/Akt and JAK STAT pathways. MiR-23a was also up-regulated by sinomenine and antisense transfection reduced sinomenine effects on these two pathways, but no target of miR-23a was identified (Xu et al., 2019). In breast cancer cells, sinomenine suppressed invasion, proliferation and also induced apoptosis by down-regulating miR-324-5p (Song et al., 2015) and up-regulating miR-29 (Gao G. et al., 2019). Down-regulation of interleukin 4 (IL-4) resulted in decreased miR-324-5p expression and, in turn, CUEDC2, its reported target gene, was up-regulated. This resulted in increased NF-kB–IkB binding, with lowered NF-kB activity. MiR-29 up-regulation resulted in increased PDCD4 expression and downstream inhibition of the JNK and MERK/ERK pathways.

**Sanguinarine** is a toxic alkaloid found in several plants of the poppy family (Papaveraceae). In gastric cancer cells, sanguinarine was found to down-regulate miR-96-5p and miR-29c-3p and subsequently increase MAP4K4, a target gene of both miRNAs, with downstream activation of MAPK/JNK pathway and decreased tumor proliferation (Dong et al., 2019). In HCC lines (Zhang B. et al., 2019), sanguinarine up-regulated miR-16-2 in a p53-dependent manner with anti-proliferative effects. Moreover, mouse HCC xenografts showed reduced tumor growth in the same study. While no direct mRNA target of miR-16-2 was explored, Bcl-2 and Cyclin D1 levels, two accepted targets of miR-16 (Cimmino et al., 2005; Liu et al., 2008), were found to be reduced.

Coptisine and dauricine are two alkaloids isolated from the Chinese goldthread (*Coptis chinensis* Franch.) and the Asian moonseed vine (*Menispermum dauricum* DC.), respectively. Both showed anti-tumor effects on hepatocellular carcinoma cells *in vitro* and *in vivo*. **Coptisine** was found to up-regulate miR-122 and lower proliferation and tumor size (Chai et al., 2018). **Dauricine** inhibited glycolysis via up-regulation of miR-199 which directly targeted and inhibited hexokinase 2 (HK2) and pyruvate kinase muscle isozyme 2 (PKM2). Treatmend with dauricine also lead to decreased proliferation and increased chemosensitivity to sorafenib, cisplatin and isoliensinine (Li W. et al., 2018).

**Berberine** is a further alkaloid found in *C. chinensis* rhizomes. This compound suppressed IL6 and STAT3 in a multiple myeloma cell line, a possible mechanism for the down-regulation of miR-21 was also observed. MiR-21 suppression was followed by PDCD4 overexpression with anti-cancer effects (Luo et al., 2014). It appears berberine also has chemosensitizing properties as shown by You et al. (2016) and Chen et al. (2015) who increased cisplatin sensitivity by up-regulating miR-203 and inhibiting miR-93 in a gastric cancer cell line and ovarian cancer cell line, respectively. You et al. revealed Bcl-w as a target for miR-203 and Chen et al. showed that berberine elicited its effects through the miR-93-PTEN/AKT pathway.

**Lycorine** is toxic alkaloid isolated from plants of the Amaryllidaceae family, which includes popular ornamental plants like snowdrops and daffodils. Lycorine was shown to decrease proliferation in a NSCLC cell population. It up-regulated miR-186, which was found to directly inhibit the cyclin-dependent kinase 1 (CDK-1) expression with subsequent destabilization of the cellular cycle (Li L. et al., 2019).

## Polysaccharides

Polysaccharides are a vast class of biogenic polymers formed by monosaccharide units connected by glycosidic bonds. These compounds consist of anywhere between 40 and 3,000 linked monosaccharides that are either connected to form linear structures or branch out into side chains (Zong et al., 2012). In plants, they are mainly involved in energy storage or they play a structural role. Many compounds have qualities that allow various medical applications. Some even have anti-cancer properties, with potential uses ranging from dietary supplements for disease prevention, to antioxidants, immunomodulators, apoptosis inducers and metastasis inhibitors (Yu et al., 2018). Polysaccharides are produced by a variety of organisms, from microorganisms (e.g., bacteria, fungi) to animals, plants and marine algae. In this section, we will only focus on the plant-derived ones, mentioning compounds which have a mechanism of action involving ncRNAs.

A homogenous polysaccharide extracted from the leaves of the tea shrub (*Camellia sinensis* (L.) Kuntze), referred to as **green tea polysaccharide** was shown to have anti-proliferative and anti-migratory effects on prostatic cancer both *in vitro* and *in vivo*. Upon treatment with green tea polysaccharide, miR-93 levels were down-regulated and the Bax/Bcl2 ratio and caspase-3 expression were increased but no direct target of miR-93 nor any potential cellular pathways were explored (Yang et al., 2019c). However, in a follow-up study, the same author group revealed disabled homolog 2 (*DAB2*) as a potential target of miR-93. Up-regulation of DAB2 would act in an inhibitory fashion on the Akt/ERK1,2 pathways, accounting for the observed anti-proliferative and anti-metastatic effect (Yang et al., 2019d).

Besides the tea shrub, another source of polysaccharides with proven anti-cancer activity is the plant species *Patrinia heterophylla* Bunge (Lu et al., 2010), which is used in TCM in a form known as Patriniae Herba. The aqueous **extract of Patrinae Herba** which most probably contains polysaccharidic compounds, showed anti-proliferative, anti-metastatic properties in hepatocellular carcinoma cell lines, via up-regulating the *lncHTR2A-AS1* and subsequently influencing the concentration of several proteins–MMP2, P21, caspase 3, E-Cadherin (Guan et al., 2020).

Promising natural products also originate from plants of the genus *Actinidia*, whose most famous representative is the vine producing the kiwifruit. Experiments using ***Actinidia eriantha*** Benth. **polysaccharide (AEP)** on a murine leukemia virus-induced tumor, showed potential immunomodulatory properties of AEP on macrophages by influencing lncRNAs (*D730047E02Rik*, *Gm14047*, *A_30_P01020139*, *A_30_P01026293* and *A_30_P01032196*), which could subsequently modulate the mRNA expression of different genes implicated in the immune response. In this study, a predicted target was the NF-κB p65 protein, which could account for the macrophagic activation observed in the cell lines (Chen X. et al., 2019).

The only compound studied in pediatric cancers was the ***Angelica sinensis* polysaccharide (AP)**. AP is a polysaccharide found in the root of *Angelica sinensis* (Oliv.) Diels, commonly known as *dong quai* or female ginseng. In a study on neuroblastoma cells, AP treatment resulted in increased apoptosis (as evidenced by higher expression of Bax, caspase-3 and -9 and lower expression of Bcl-2) and reduced migration and proliferation (Yang J. et al., 2018). MiR-675 levels were down-regulated and transfection with a miR-675 mimic reduced the effects of AP. Mechanistic studies were performed and miR-675 was shown to up-regulate the PI3K/AKT and JAK/STAT pathways, and AP down-regulated these pathways by down-regulating miR-675. Another study treating neuroblastoma cells with AP revealed the same pro-apoptotic and anti-proliferative effects already described and showed that the PI3K/AKT and ERK1/2 pathways were inhibited. MiR-205 was up-regulated and, after transfection with a miR-205 inhibitor, the effects of AP were reduced (Yang et al., 2019b). MiR-205 was shown to target the ZEB1 gene which is known to induce EMT in various cancers (Eger et al., 2005).

The Mongolian milkvetch (*A. membranaceus*) does not only yield the astragalosides discussed in the saponins section, but also a mixture of polysaccharides that were shown to have anti-proliferative effects. Chu et al. treated osteosarcoma cell lines with **Astragalus polysaccharide (APS)**, resulting in up-regulation of miR-133a, which in turn led to decreased expression of several proteins of the JNK pathway, a member of the MAPK pathway, implicated in cell proliferation and apoptosis. In this study, the authors observed that APS also promoted cell cycle arrest in the S phase and apoptosis, and the anti-migratory effect was reflected by lower measured levels of MMP-2 and -9 (Chu et al., 2018).

There is an additional plant extract worth mentioning that has been shown to contain compounds from various classes (phenols, flavonoids, triterpenes) along with polysaccharides. It is the **Spica Prunellae extract (SPE)**, obtained from *Prunella vulgaris* L., a plant commonly known by the very fitting names of self-heal or heal-all. In a study on colon cancer cell lines, treatment with SPE showed anti-proliferative and apoptosis inducing properties via up-regulation of the miR-34a which, in turn, was shown to decrease the expression levels of Notch1,2 and Bcl-2 (Fang Y. et al., 2017). Moreover, in a different study using bioinformatic analysis, the same extract showed 5-FU chemo-sensitizing properties in colon cancer cell lines, supposedly via up-regulation of miR-494, which could down-regulate TOP 2α expression, an enzyme involved in DNA transcription (Fang et al., 2019).

## Organosulfur Compounds

Organosulfur compounds are a chemically heterogeneous group of organic molecules that contain sulfur. In human diets, two major sources of organosulfur compounds are represented by vegetables from Alliaceae (garlic, onions, leaks etc.) and Brassicaceae (cabbage, Brussels sprouts, kale, broccoli, cauliflower, watercress etc.) families. These organosulfur compounds, such as alliin (onions) or glucosinolates (*Brassica*-species), protect plants from pathogens and insects. A side aspect is that these compounds are key for the taste of the plant-based foods. Moreover, they have health-promoting effects as part of the human diet. In a 2016 review by Biersack (2016) the interplay between three major organosulfur compounds and miRNAs in adult anti-cancer therapy was precisely described. However, over the last four years, more insights were gained, with some few studies also focused on pediatric-age cancers.

**Diallyl disulfide (DADS)**, a compound found in garlic and other plants in the genus *Allium*, showed anti-proliferative effects in gastric cancer cell lines and tumor bearing mice via up-regulation of miR-200b and miR-22 which directly target the Wnt1 pathway (Tang et al., 2013). Likewise, in a different study on gastric cell lines, DADS up-regulated miR-34a, which, in turn, led to down-regulation of p-PI3K and *p*-Akt, resulting in apoptosis induction and invasiveness reduction (Wang G. et al., 2016). In a different study employing breast cancer cell lines and tumor bearing mice, DADS also increased miR-34a expression and was shown to directly target c-Src, affecting its downstream targets Ras/ERK. This led to reduced proliferation and invasiveness *in vitro*, as well as to reduced tumoral volume in mice models, compared to the control groups (Xiao et al., 2014).

A further agent with potent anti-cancer activity is **sulforaphane (SFN)**, an organosulfur compound found in many vegetables of the Brassicaceae family. SFN showed varied anti-cancer properties, ranging from cancer cell stemness suppression, anti-proliferation and apoptosis induction to metastasis and EMT inhibition and can be used as a chemo-sensitizing co-agent in a wide range of cancers.

In a study on two colon cancer cell lines, SFN treatment has effectively decreased miR-21 and human telomerase reverse transcriptase (hTERT) mRNA levels, by means of epigenetic regulation showing anti-proliferative and apoptosis-inducing effects (Martin et al., 2018). In another recent study on colon cancer cells, the co-treatment of SFN and a miR-15b-5p inhibiting agent, showed very potent anti-apoptotic properties, compared to the two substances alone (Gasparello et al., 2020). In a study on two gastric cancer cell lines and a control cell line, SFN dose-dependently increased miR-9 and miR-326 expression in one cell line. These miRNAs targeted and reduced the expression of the caudal type homebox 1 and 2 (CDX1, CDX2) protein levels, which are known to act as modulators of intestinal stem cell differentiation. The observed phenotypic effect was apoptosis induction (Kiani et al., 2018).

In pancreatic cancer, one study done on different cell lines showed that SFN induced miR-135b-5p expression which, in turn, binds to the 3′UTR region of the Rat sarcoma (RAS) Protein Activator Like 2 (*RASAL2*) gene, leading to its up-regulation, having an anti-proliferative effect *in vitro*. These findings mirror the expression levels found in tumor tissues compared to healthy tissue (Yin et al., 2019a). In a different *in vitro* study on pancreatic cancer, SFN alone or in combination with other natural compounds (quercetin and green tea catechins), were shown to have anti-proliferative effects through up-regulation of the miRNA let-7 and subsequent down-regulation of its target K-Ras (Appari et al., 2014). In a different study miR-365a-3p was significantly underexpressed in pancreas cancer tissue compared to healthy pancreas tissues. The group showed the ability of SFN to induce miR-365a-3p expression and confirmed c-Rel as target, known to act on NF-κB signaling. In this study, SFN treatment showed anti-metastatic and apoptosis-inducing properties *in vitro*, confirmed by decreased tumor size in xenograft models (Yin et al., 2019b).

Interestingly, the chemoresistance of pancreatic cancer might be partly owed to a decreased intercellular communication through gap junctions and reflected in low connexin 43 (Cx43) expression. In one study on pancreatic cancer cells, SFN treatment inhibited miR-30a-3p expression, which was shown to target Cx43 and increase its expression, which led to better gemcitabine chemosensitivity (Georgikou et al., 2020).

Lung cancer stem cells showed high expression rates of miR-19a and mir-19b, which were significantly decreased by SFN treatment. These miRNAs were shown to target GSK3β, which affected the downstream Wnt/β-catenin pathway. This way, SFN showed stemness suppression, alteration of tumor sphere formation and resulted in apoptosis induction (Zhu et al., 2017). In a different study on NSCLC, SFN decreased the expression of a different miRNA, miR-616-5p which was then shown to also target GSK3β and influence the aforementioned pathway. In this study, treatment with SFN was effective in suppressing EMT and reducing metastatic potential (Wang D. X. et al., 2017). The SFN treatment in NSCLC cell lines was shown to also up-regulate miR-214 expression, which was shown to target the c-Myc proto-oncogene, leading to stemness inhibition, apoptosis induction and to better cisplatin chemosensitivity *in vitro* as well as *in vivo* (Li et al., 2017).

In an *in vitro* study on bladder cancer, SFN induced the expression of miR-200c which, in turn, targeted ZEB1, leading to increased E-cadherin expression, suggesting that SFN acts as a TME modulator in this type of cancer (Shan et al., 2013). In nasopharyngeal carcinoma, SFN increased miR-124-3p levels, which down-regulated STAT3, resulting in decreased proliferation and apoptosis inducement (Li X. et al., 2018). In a different study on gastric cancer cell lines, SFN also up-regulated miR-124-3p. This miRNA in turn targeted IL-6R and STAT3 which resulted in better response to cisplatin treatment, especially by counteracting the stemness-inducing properties of the treatment with cisplatin alone (Wang X. et al., 2016).

A study on AML showed that sulforaphane might reduce the pathologically up-regulated miR-155, and hereby induce differentiation and apoptosis, however, there is no mechanistic explanation of this effect (Koolivand et al., 2018). In GBM cell lines, SFN has been shown to repress the Wnt/β-catenin/TCF4 pathway and indirectly decrease the miR-21 expression. This down-regulation then altered the BAX/BCL-2 ratio and increased caspase 4 and 7 levels, with pro-apoptotic consequences. This effect was especially useful in the Temozolomide treated cells, acting as chemosensitizer (Lan et al., 2015).

Effects related to lncRNAs were reported by a study which investigated a prostatic cancer cell line and compared it to a non-cancerous control before and after SFN treatment using whole genome RNA sequencing. Significant differences in expression of about 100 lncRNAs involved in cancer metabolism, cell cycle regulation and signal transduction were noted. Moreover, the suppression of *LINC01116* through SFN showed anti-proliferative effects by modulating expression of several genes implicated in metabolism (*GADPH*), autophagy (*MAP1LC3B2*) and regulation of chromatin structure (H2AFY). The authors hypothesized that this LINC01116 could modulate target genes expression by forming ssRNA:dsDNA triplexes with them (Beaver et al., 2017).

Lastly, another organosulfur compound found in Brassicaceae, **phenethyl isothiocyanate (PEITC)** has been shown to increase the expression of miR-194 in prostate cancer cell lines. This miRNA, in turn, targets and decreases the expression of the bone morphogenic protein 1 (BMP-1), resulting in decreased levels of MMP2 and 9 which are associated with a lesser migratory potential (Zhang C. et al., 2016).

**FIGURE 3:**
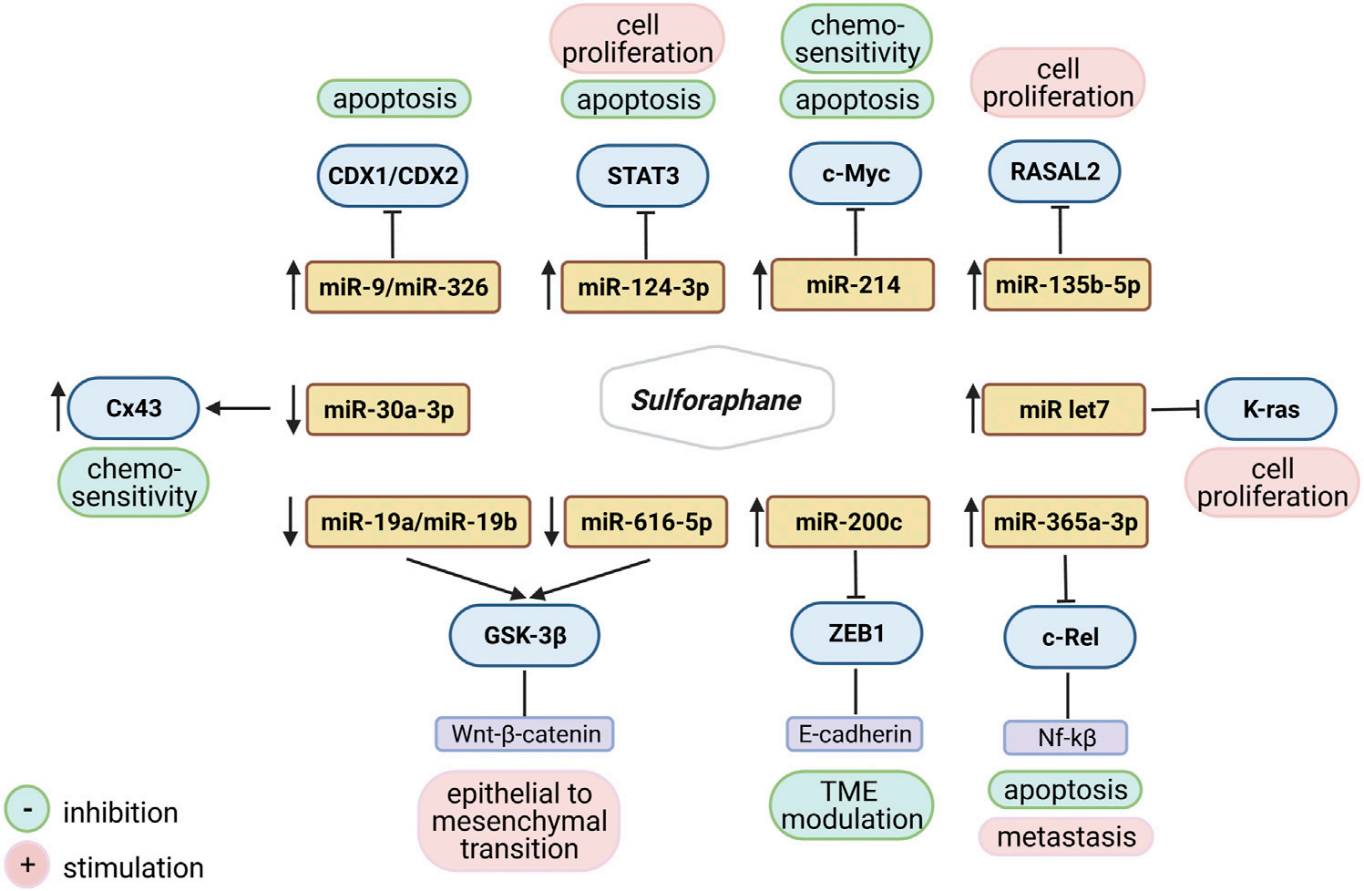
The mechanism of action of sulforaphane by modulating expression of several miRNAs (yellow rectangles) which target specific molecules (blue ovals) and/or pathways (purple rectangles), inducing the observed phenotypical changes–inducing of apoptosis, inhibition of cell proliferation, inhibition of metastasis, inhibition of EMT, TME modulation and inducing of chemosensitivity.

The presented findings regarding organosulfur compounds are summarized graphically in Figure 3.

**FIGURE 4:**
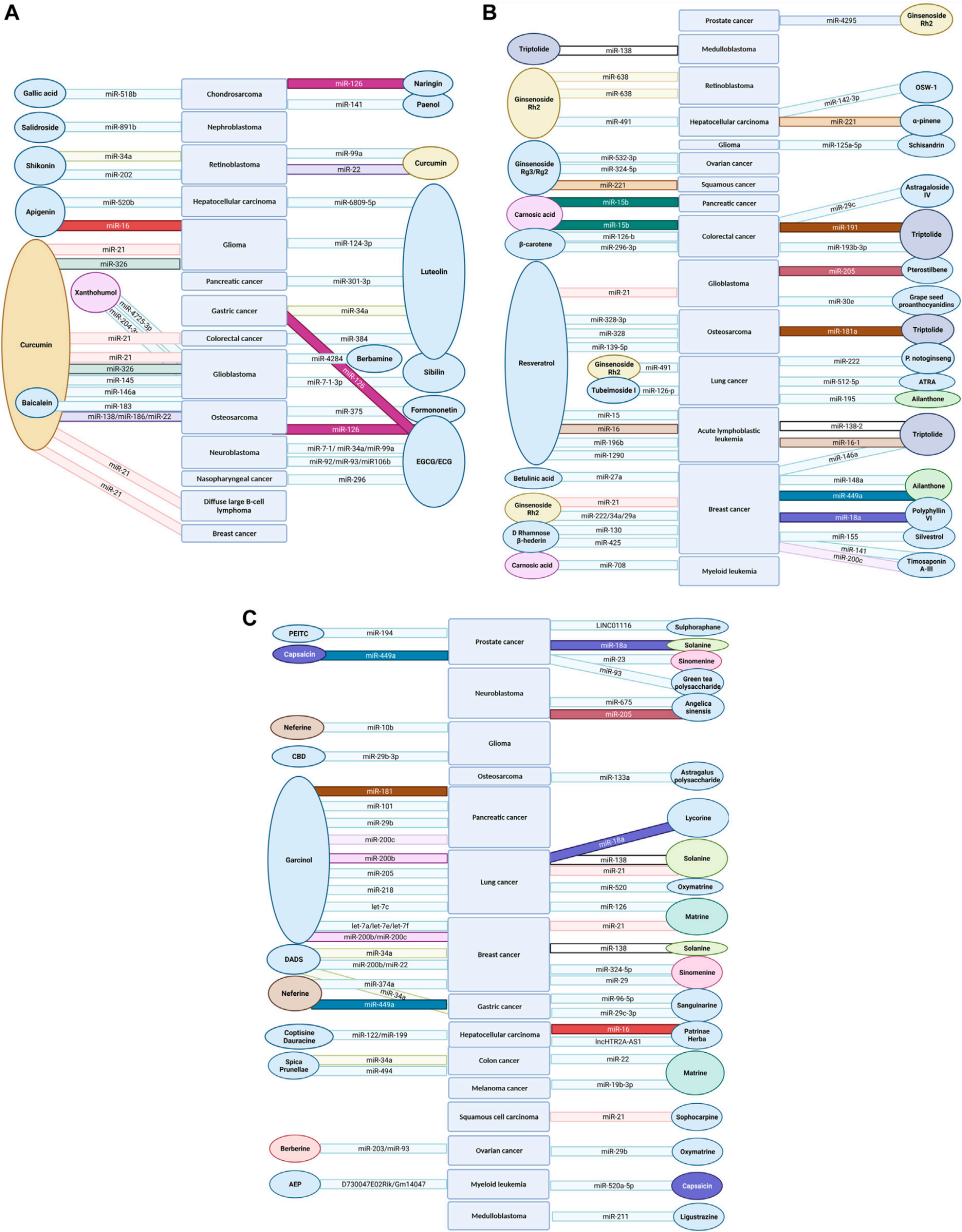
Summarized in this figure are natural compounds (ovals), which influence specific cancers (big rectangles) through specific ncRNAs (long rectangles): **(A)** phenolic acids and other monophenols, flavonoids and curcuminoids; **(B)** tannins, terpenoids, saponins, stilbenoids, carotenoids (except for ATRA, already presented in Figure 2); and **(C)** polyprenylated acylphloroglucinols, cannabinoids, alkaloids, polysaccharides and organosulfur compounds.

Summing up, in this entire segment, we presented natural compounds from different chemical classes and reviewed their ncRNA-dependent anti-cancer effects. In order to visualize the most important information presented in the main body of this review, we created a table that lists most of the cancer types discussed herein, along with two or more examples of natural compounds studied for each cancer, the ncRNAs which are known to be involved in each particular case and, wherever available, with their downstream targets (Table 2). Additionally, Figure 4 summarizes our review in a graphical manner: phenolic acids and other monophenols, flavonoids and curcuminoids in Figure 4A; tannins, terpenoids, saponins, stilbenoids and carotenoids (except for ATRA, already presented in Figure 2) in Figure 4B; polyprenylated acylphloroglucinols, cannabinoids, alkaloids, polysaccharides and organosulfur compounds in Figure 4C.

**TABLE 2:** Types of cancers covered in this review and some of the most representative examples of natural compounds/derivatives acting on ncRNAs, the type of material studied on (cell lines, i.e., *in vitro* or mouse models i.e., *in vivo*), the mechanistic pathway downstream of the ncRNA and the phenotypic effect achieved.

*Cancer*	Natural compound	*In vitro* ^1^/*In vivo* ^2^	ncRNA	Targets and/or downstream effectors	Phenotypic effects^a^	Reference
Hepatocellular cancer	Apigenin	1	miR-520b	ATG7	CS	Gao et al. (2018)
	Dauricine	1, 2	miR-199a	HK2, PKM2	A, CS, E	Li W. et al. (2018)
Gastric cancer	Luteolin	1	miR-34a	BCL-2	A	Wu et al. (2015a)
	Sulforaphane	1	miR-124–3p	IL-6r, STAT3	CS	Wang X. et al. (2016)
Colorectal cancer	Astragaloside IV	1	miR-29c	B7-H3	P	Wang et al. (2018)
	Astragaloside IV	1	miR-134	CREB1	EMT, CS	Ye et al. (2017)
Pancreatic cancer	Luteolin	1	miR-301–3p	Caspase-8	P	Moeng et al. (2020)
	Sulforaphane	1, 2	miR-135b-5p	RASAL2	P	Yin et al. (2019a)
Non small-cell lung cancer	Garcinol	1	miR-200b let7	E-cadherin, vimentin, ZEB1	EMT, CS	Farhan et al. (2019)
	ATRA	1	miR-512-5p	p21	A, E	Chu et al. (2016)
Oral squamous cell cancer	Sophocarpine	1, 2	miR-21	PTEN, p38/MAPK	P, A, EMT	Li W. et al. (2017)
	Ginsenoside Rg3	1, 2	miR-221	TIMP3, PI3K/AKT, MAPK/ERK	P, EMT	Cheng and Xing (2019)
Nasopharyngeal cancer	EGCG	1	miR-296	STAT3	M	Lin et al. (2020)
	Sulforaphane	1	miR-124–3p	STAT3	P, A, TME	Li X. et al. (2018)
Prostatic cancer	Sulforaphane	1	LINC01116	GADPH, MAP1LCD3B H2AFY	E, GS, A	Beaver et al. (2017)
	PEITC	1	miR-194	BMP-1 MMP2, -9	M	Zhang C. et al. (2016)
Breast cancer	Betulinic acid	1	miR-27a	ZBTB10, Sp-1, -3, -4	P, A, AG	Liu et al. (2012); Mertens-Talcott et al. (2013)
	Ailanthone	1	miR-148a	CyclinD1, p53 and p21 Caspase-3, -9 AMPK, Wnt/β-catenin	P, M	Gao W. et al. (2019)
Neuroblastoma	*Angelica sinensis* polysaccharide	1	miR-675	KIF1Bβ, CD44, lncRNA-H19	P, A, M	Yang J. et al. (2018)
	EGC, EGCG	1	miR-7-1		A	Chakrabarti et al. (2013)
CML	Capsaicin	1	miR-520a-5p	STAT3	P, A	Kaymaz et al. (2014)
AML	Ginsenoside Rh2	1,2	miR-21	BCL-2	A	Wang and Wang (2015)
Osteosarcoma	Resveratrol	1	miR-139-5p	NOTCH1	A	Xiao et al. (2020)
Nephroblastoma	Triptolide	1	miR-193b-3p	KLF4, PI3K/AKT, ERK	A, M	Hang et al. (2019)
	Salidroside	1	miR-891b	Cyclin D1, MMP-2, vimentin, p53, p21, caspase-3, -9 PI3K/AKT/mTOR	P, A, M	Li H. et al. (2019)
Retinoblastoma	Curcumin	1	miR-22	Erbb3	P, M	Sreenivasan et al. (2012)
	Shikonin	1	miR-34a, miR-202	MYCN	P	Su et al. (2018)
Medulloblastoma	Ligustrazine	1	miR-211	PI3K/AKT and mTOR	P, M	Xu D. et al., 2018
	Triptolide	1	miR-138	CDK6	P, M	Zhang H. et al., 2018
Glioma/GBM	Xanthohumol	1	miR-4725–3p	STIM1	P, EMT	Ho et al. (2018)
	Apigenin	1	miR-16	BCL-2 - NFkB/MMP9	A	Chen X. J. et al. (2016)
	Resveratrol	1	miR-21	IκB/p50-p65	P	Li H. et al. (2013)
	BBMD3	1	miR-4284	JNK/AP-1	A	Yang F. et al. (2014)

a **A**poptosis; **A**, Inhibition of **A**ngio**g**enesis; **AG**, Anti-**p**roliferation; **P**, Inhibition of **m**etastasis; **M**, Inhibition of **E**pithelial to **m**esenchymal **t**ransition; **EMT**, Regulation of **t**umoral **m**icro**e**nvironment; **TME**, Regulation of cellular **e**nergetics; **E**, Promotion of **g**enomic **s**tability; **GS**, Inducing **c**hemo-**s**ensitivity; **CS**.

## Future Perspectives

In this review, we summed up recent findings related to anti-cancer plant-derived natural compounds that have a ncRNA (mostly miRNA)-related mechanism of action. In our search, several points raised our attention.

First, most studies were *in vitro* analyses, using cancer specific cell lines and researching the effects of treatment with the specific natural compound, compared to treatment with a negative control substance. Hence, only exceptionally the effect of a natural compound was compared to an already proven anti-tumorigenic substance. Expression levels of specific ncRNAs were then measured via microarray or PCR. Likewise, levels of potential mRNA targets and/or the corresponding proteins were subsequently analyzed. In some cases, direct targeting was confirmed via luciferase reporter assay. Rescue assays, to ensure that the noted effects were indeed induced by the studied substances via ncRNA and their downstream pathways were rarely performed. Few studies also confirmed the finding *in vivo*, using tumor-bearing mice models. Unfortunately, this thorough analysis was not conducted in all studies and we want to point out that in order to introduce these substances into clinical practice, comprehensive mechanistic studies are necessary.

**FIGURE 5:**
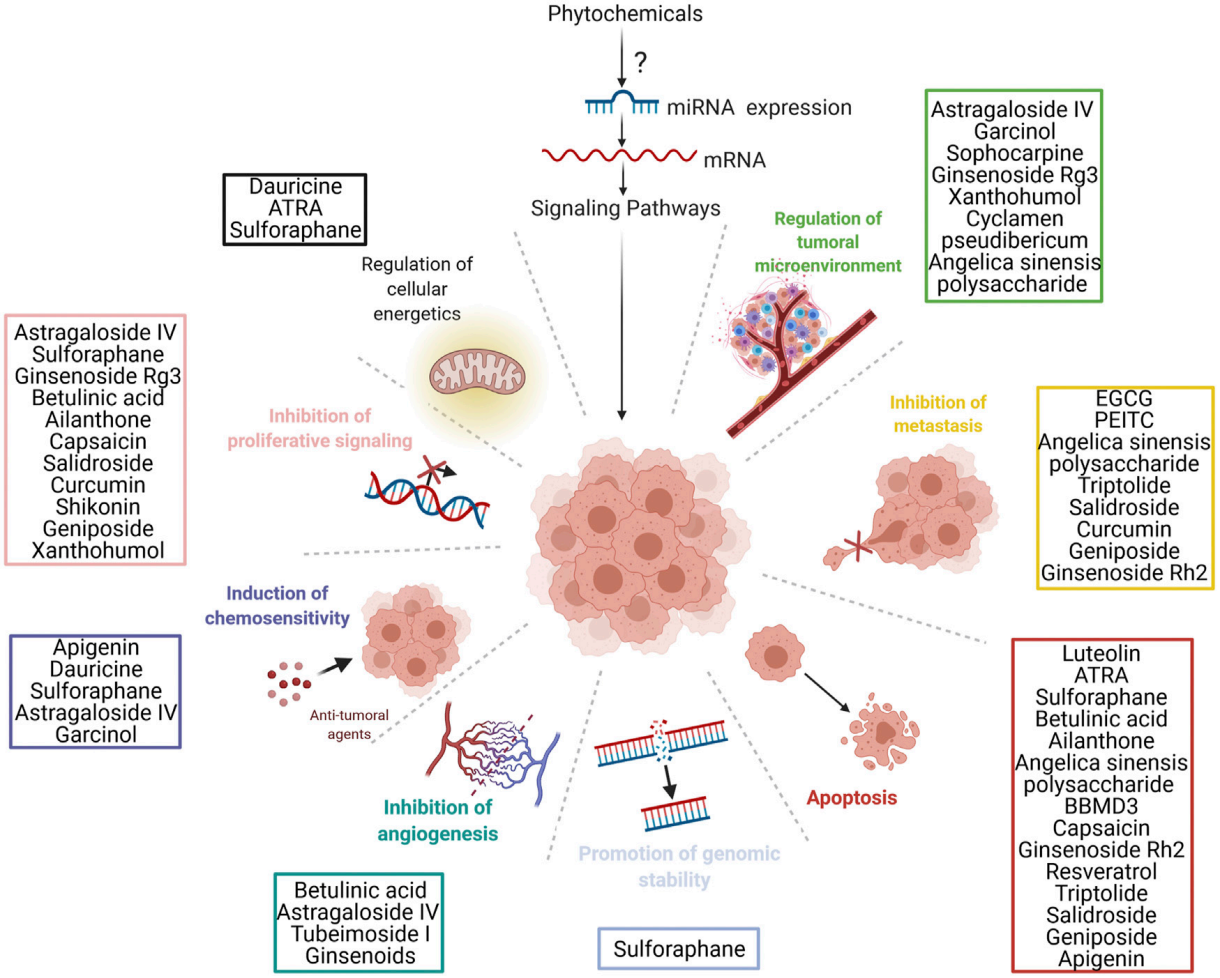
The general mechanisms by which plant-derived natural compounds (phytochemicals) influence miRNA expression. In turn, these miRNAs can alter the expression of different proteins implicated in several cancer signaling pathways by binding to target mRNA. This alteration at the molecular level then leads to the phenotypical changes observed *in vitro* and *in vivo*. The major phenotypical changes are represented in the figure. Lastly, the major types of natural compounds and their pathways of action are summarized in the figure.

Second, we observed that natural compounds can have heterogenous anti-cancer effects by restoring dysregulated ncRNA expression levels and, in turn, indirectly targeting numerous downstream molecules of ncRNAs. Natural compounds can act as anti-proliferative, apoptosis-inducing and anti-metastatic agents, but can also influence the TME, the energetic metabolism, angiogenesis and lastly, induce chemosensitivity (Figure 5). An important limitation of the studies we analyzed is the fact that only exceptionally the mechanism through which the natural compound regulates the ncRNA expression was researched. We can envision several possibilities: the natural compound directly/indirectly regulates the transcription of the ncRNA; the natural compound inhibits the biogenesis of ncRNAs, in a similar way to small-molecule inhibitors of miRNA (Vo et al., 2014); or the natural compound binds the mature form of ncRNA prolonging its half-life or accelerating its degradation. Apart from these putative mechanisms, many of the reviewed compounds are known to have effects on membrane fluidity and permeability (Tsuchiya, 2015). As already described for terpenoids, this could account for certain, presumably unspecific effects of phytochemicals on miRNA function by influencing membrane association of AGO proteins. Deciphering these aspects is of great importance in order to understand the mechanism of action of natural compounds on ncRNAs in cancer and could help in improving the efficacy of semi-synthetic natural compound analogs or derivatives.

Third, we only focused on pharmacological products that where directly derived from natural compounds, excluding those synthetically designed based on natural compounds. However, there are compounds derived from plants, which are further synthetically adjusted (for example ATRA), which have shown solid anti-oncogenic capacities and some of which are also clinically used. This observation clearly outlines that after the initial confirmation of the anti-cancer effect of natural compounds, subsequent synthetic modifications that improve the pharmacokinetics and pharmacodynamics of the compound are necessary in order to translate the findings into clinical practice. Of note, in order to purposefully make synthetic modifications that improve the pharmacodynamics of the natural compound, it is crucial to understand its biomolecular mechanism of action. Therefore, in-depth mechanistic studies are highly necessary.

Fourth, we noticed that some of the reported substances were used in phase I-III clinical trials, with sometimes inconclusive and sometimes favorable results. While there are several phase I trials that proved the therapeutic efficacy of natural compounds, only few phase II trials reported favorable outcomes. Many of the phase II clinical studies were terminated due to lack of safety, funding, or lack of efficacy. Nevertheless, there are also a few phase III clinical trials that are either completed or currently recruiting. Data regarding some very popular compounds that potentially also function via ncRNAs is already available. A phase II clinical study on curcumin showed beneficial effects on reducing radiation-induced dermatitis in breast cancer patients (Ryan et al., 2013). A phase III study on the same topic was completed by the same study group, but no results have been published (**NCT01246973**). In contrast, a phase II clinical study on resveratrol (SRT501) in combination with bortezomib on refractory multiple myeloma patients showed no efficacy and several concerning adverse side effects (nausea, vomiting, and renal failure), having an unacceptable safety profile (Popat et al., 2013). Two phase III clinical trials regarding treatment with curcumin in prostatic cancer are currently recruiting. One of them measures the progression of low-risk prostatic cancer (**NCT03769766**), while the other assesses recurrence post radical prostatectomy (**NCT02064673**). A phase II clinical trial on curcumin in advanced inoperable pancreatic cancer has been completed in 2020, results are still to be published (**NCT00094445**). Unfortunately, only scarcely do these studies report mechanistic analyses regarding the anti-tumorigenic effect of the natural compounds. We can only speculate that natural compounds function in clinical setting via ncRNAs. Meanwhile, methodological tools for investigating molecular interactions of natural compounds with ncRNAs are readily available. For instance, we highly recommend the analysis of ncRNA expression on tumor biopsies after neoadjuvant treatment/palliative treatment with natural compounds and/or the analysis of the circulating ncRNAs from the different bodily fluids before and after treatment with natural compounds.

The future and the role of natural compounds in medicine and more precisely in oncology remains undetermined. In a recent critical publication Grollman and Marcus (2020) presented the historical development and the current *status quo* regarding the use of “botanical medicine”. Mainly, the use of herbal remedies is rooted in ignorance or lack of knowledge regarding potential side effects or rejection of evidence-based medicine, especially in western societies. Most concerningly, the authors emphasized the complex financial interests regarding regulation of dietary supplements. Funding of the WHO through the Chinese government, lack of regulation regarding safety profiles, the planned introduction of TCM and alternative medicine in the 2021 ICD-11 are all current critical problems and raising awareness regarding this topic is essential. While the authors emphasized the hazardous effects in the unregulated use of “herbal remedies”, the intrinsic effects and potentiality of these compounds is presented in many of the studies mentioned above. That being said, we do not advocate for the uncontrolled use of such compounds as “health boosters”. We do support, however, an evidence-based approach in the use of such compounds mainly as adjuvant treatments in specific cancer types where the effect is proven, with acceptable safety profiles documented in phase III and IV clinical trials. Surely, many further mechanistic investigations regarding such compounds are needed until official clinical approval of one such substance will take place.

Taking a look toward the future, we would like to conclude with noting the auspicious coexistence of three aspects: the overwhelming diversity of chemical compounds produced throughout the plant kingdom, the accumulating evidence linking several such compounds to ncRNA-mediated anti-cancer effects and the availability of the proper methodological tools for investigating their exact mechanism of action. In this combination, we recognize promising circumstances for the development of reliable, evidence-based cancer treatments starting from natural compounds. There are good reasons to expect that scientific efforts invested in this topic, while still abundantly needed, will eventually prove to be worthwhile.
